# Transcription through the eye of a needle: daily and annual cyclic gene expression variation in Douglas-fir needles

**DOI:** 10.1186/s12864-017-3916-y

**Published:** 2017-07-24

**Authors:** Richard Cronn, Peter C. Dolan, Sanjuro Jogdeo, Jill L. Wegrzyn, David B. Neale, J. Bradley St. Clair, Dee R. Denver

**Affiliations:** 1 Pacific Northwest Research Station, USDA Forest Service, Corvallis, OR 97331 USA; 2 University of Minnesota – Morris, Morris, MN 56267 USA; 3 Department of Integrative Biology, Oregon State University, Corvallis, OR 97331 USA; 4 Department of Ecology and Evolutionary Biology, University of Connecticut, Storrs, CT 06269 USA; 5 Department of Plant Sciences, University of California – Davis, Davis, CA 95616 USA

**Keywords:** Circannual, Diurnal, Dormancy, Douglas-fir, *Pseudotsuga menziesii*, RNA-Seq, Transcriptome

## Abstract

**Background:**

Perennial growth in plants is the product of interdependent cycles of daily and annual stimuli that induce cycles of growth and dormancy. In conifers, needles are the key perennial organ that integrates daily and seasonal signals from light, temperature, and water availability. To understand the relationship between seasonal cycles and seasonal gene expression responses in conifers, we examined diurnal and circannual needle mRNA accumulation in Douglas-fir (*Pseudotsuga menziesii*) needles at diurnal and circannual scales. Using mRNA sequencing, we sampled 6.1 × 10^9^ reads from 19 trees and constructed a de novo pan-transcriptome reference that includes 173,882 tree-derived transcripts. Using this reference, we mapped RNA-Seq reads from 179 samples that capture daily and annual variation.

**Results:**

We identified 12,042 diurnally-cyclic transcripts, 9299 of which showed homology to annotated genes from other plant genomes, including angiosperm core clock genes. Annual analysis revealed 21,225 circannual transcripts, 17,335 of which showed homology to annotated genes from other plant genomes. The timing of maximum gene expression is associated with light intensity at diurnal scales and photoperiod at annual scales, with approximately half of transcripts reaching maximum expression +/− 2 h from sunrise and sunset, and +/− 20 days from winter and summer solstices. Comparisons with published studies from other conifers shows congruent behavior in clock genes with Japanese cedar (*Cryptomeria*), and a significant preservation of gene expression patterns for 2278 putative orthologs from Douglas-fir during the summer growing season, and 760 putative orthologs from spruce (*Picea*) during the transition from fall to winter.

**Conclusions:**

Our study highlight the extensive diurnal and circannual transcriptome variability demonstrated in conifer needles. At these temporal scales, 29% of expressed transcripts show a significant diurnal cycle, and 58.7% show a significant circannual cycle. Remarkably, thousands of genes reach their annual peak activity during winter dormancy. Our study establishes the fine-scale timing of daily and annual maximum gene expression for diverse needle genes in Douglas-fir, and it highlights the potential for using this information for evaluating hypotheses concerning the daily or seasonal timing of gene activity in temperate-zone conifers, and for identifying cyclic transcriptome components in other conifer species.

**Electronic supplementary material:**

The online version of this article (doi:10.1186/s12864-017-3916-y) contains supplementary material, which is available to authorized users.

## Background

The sensing of daily, seasonal, and annual environmental variation in land plants is accomplished using a diverse array of organs, transcriptional regulators that drive oscillatory functions, and pathways that refine, optimize, and entrain metabolic cycles to cyclic environmental stimuli. Circadian patterns are ubiquitous in photosynthetic [1–4] and non-photosynthetic [5, 6] organisms, and they are essential for coordinating external signals for optimally timing transcriptional activity to match the demands of growth and phenology with resource availability. The genetic basis for diurnal responses in model plants have been intensively studied using mutant screens [4, 7, 8] and global transcription [7], and these studies have identified components of the central oscillator (“core clock”) and genes that are targets of cyclic activation and repression. Variability in core clock genes alters the timing of the clock and clock-dependent pathways, so subtle changes in genes controlling clock cycles may contribute to local adaptation on a short time scale, and evolutionary divergence on a longer time scale [1, 9].

In temperate zone trees, circadian cycles are superimposed on longer annual cycles involving transitions between active growth -- the time when light energy is captured and converted into growth -- and dormancy, the time when growth potential is arrested to protect cells from seasonal stresses of cold temperatures, freezing and desiccation [10–12]. Accurately timed transitions between growth and dormancy are essential for adapting to variable environments [13] and they depend on: (1) reliable environmental cues that forecast future change; (2) diverse sensory organs; and (3) competing biochemical networks that integrate sensory signals and shift responses from one state (growth; dormancy; senescence) to the next. Photoperiod and light quality are known to be important cues for initiating seasonal growth rhythms and establishing the onset of dormancy for many trees [11, 12, 14, 15], so pathways involved in light capture and photoperception are expected to show annual rhythmic variation. As dormancy is established, trees also sense and respond to cold temperatures (chilling units) by increasing cold hardiness and increasing resistance to desiccation. After chilling thresholds are met, trees respond to warming temperatures (forcing units) by releasing tissues from dormancy, remaining in a ‘stand-by’ state until requirements for initiating growth (heat, water availability, light quality [10, 14, 16]) are met. Genes implicated in dormancy and resumption of spring growth should also show annual cyclic variation, and these include components of the circadian clock and photoperiod-responsive genes [11, 14, 15, 17], and pathways involved in temperature and water perception [15, 18, 19], hormone regulation and cell growth [10, 20], and glucan hydrolysis [20].

The organs responsible for sensing and integrating external stimuli -- leaves, shoots, and roots -- are common to perennial plants, but signal perception and integration during the dormant season is likely accomplished by different means in conifer trees with perennial leaves (“needles”), versus trees with deciduous leaves. Perennial needles confer advantages by preserving annual investments in carbon fixation, conducting photosynthesis earlier and later in spring and fall in cold climates (e.g., northern latitudes; high altitudes) [21], or even year-round in the case of milder maritime climates [22, 23]. They offer alternative mechanisms for preventing winter embolisms [24], and providing an environmentally-responsive sensor that adds to bud- and stem-associated signals during dormancy. Perennial needles come with fitness trade-offs because they can be damaged by cold during entry into dormancy and during de-acclimation in spring [23, 25], and by photosystem excitation that can lead to the formation of reactive oxygen species during dormancy [23, 26]. Mature perennial needles can be difficult to compare at the molecular level to annual angiosperm leaves because gene expression in annual leaves changes primarily as a function of developmental state of the leaf (e.g., emergent; expanding; mature; senescent; [27]). In contrast, when conifer leaves are mature but have not started to senesce, gene expression – in theory – is only needed (1) when the protein composition of the leaf should be changed due to changes in environmental conditions, or (2) to replace proteins that have been degraded [27]. Given the complexity involved in orchestrating growth and stasis over annual cycles, annual gene expression variation in conifer needles should show high complexity and a strong relationship to changing environmental conditions, especially as compared with the annual leaves characteristic of model trees (*Populus* L.) and herbs (*Arabidopsis* Heynh. in Holl & Heynh).

To date, patterns of cyclic gene expression in conifer needles have been examined from a limited number of species. The behavior of select ‘core clock’ genes has been characterized in Norway spruce (*Picea abies* L. Karst.) under a variety of short- and long-day light regimes [28–30], and Japanese cedar (*Cryptomeria japonica* (L.f.) D.Don; [31]) under summer and winter field conditions. In these studies, most well-studied circadian genes behave in a manner similar to that described for homologs in angiosperm models, although there are notable exceptions with homologs of *flowering locus T* (*FT*) [14, 28] and *late elongated hypocotyl* (*LHY*) [30]. The most striking characteristic of gymnosperms core clock genes is that circadian cycling activity is arrested when conifers are moved to constant light or dark [29, 32]; circadian rhythms may also cease during the winter dormant period [31]. At a larger temporal scale, several studies have focused on transcriptional changes over one to several months. For example, Hess et al. [33] recently examined transcript accumulation in Douglas-fir (*Pseudotsuga menziesii* (Mirb.) Franco) using RNA-seq, with the goal of identifying transcripts responsive to sample date and environmental factors (photoperiod; maximum temperature; soil available water) during the summer growing season. Their study found that ~80% of predicted protein encoding transcripts showed significant variation by sample date. In contrast, Holliday et al. [34] examined transcript accumulation in Sitka spruce (*Picea sitchensis* (Bong.) Carr.) by microarrays, with the goal of identifying genes that were differentially expressed in the autumn months leading to winter dormancy (August – December). Their study found that a small proportion of putative genes (2224, or ~10% of those assayed) showed significant variation by sample date during the transition from fall to winter dormancy. At present, the larger annual (“circannual”) pattern of transcription has yet to be defined for any conifer.

To provide a finer-resolution understanding of the complexity of circadian and circannual cycles of gene expression in conifers, we examined gene expression in needles from Douglas-fir for cyclic expression patterns that show daily and annual periods. Douglas-fir is related to model conifers like Norway spruce and Loblolly pine (*Pinus taeda* L.) through divergence in the Early Cretaceous ~130 MYA [35], and to angiosperms like *Populus* and *Arabidopsis* through a more ancient divergence ~300 MYA. Douglas-fir has an expansive native range in North America [36], and it is noteworthy among conifers for its significant population variation in needle cold hardiness, phenology, and growth traits [37, 38]. Some of the strongest associations between provenance source and quantitative traits in conifers are exhibited by Douglas-fir needle traits related to annual cycles of freeze-avoidance, such as spring and fall needle cold hardiness, and cyclic cues that define the onset of winter like the first winter freeze and variability in the frost free period [25, 39, 40].

In this study, we use next-generation mRNA sequencing to produce individual de novo needle transcriptomes from 19 Douglas-fir trees, and use the resulting “pan-transcriptome” as a reference for mapping RNA-seq reads from experiments evaluating diurnal variation over two daily cycles and circannual variation over one annual cycle. We specifically searched for transcripts exhibiting cyclic expression [41, 42] to define the timing of maximum expression (“phase”) and the amplitude. Our results provide a detailed characterization of daily and year-round transcriptome activity in leaves from a long-lived perennial tree, and they identify a core set of transcripts that show evidence for significant cycling on daily and annual scales. Our results show congruence with other microarray- and RNA-seq-based studies of diurnal and seasonal gene expression in conifers [31, 33, 34], and they underscore the potential for using gene expression information from Douglas-fir as a baseline for examining cyclic transcriptome responses, and gene expression patterns responding to different environmental cues in temperate-zone conifers.

## Methods

### Plant materials and sample information

Trees used for annual analysis are from a larger reciprocal translocation study [19, 43] that includes multiple sources of *Pseudotsuga menziesii var. menziesii* from the Pacific Northwest of North America. Trees were chosen to maximize differences in source climatic and phenology [38]; included are families from regions that derive from cold/wet sources (47.3° N, −121.6° W, 950 m elevation), cool/wet sources (47.2° N, −123.9° W, 111 m elevation), and warm/dry sources (43.3° N, −123.1° W, 429 m elevation; Additional file 1). Two-year old trees were planted in a warm/dry region (Central Point, Oregon, USA; 42.3° N, −122.9° W, 390 m elevation) in November, 2009, and sampling of the 2010 cohort of needles was initiated on October 27, 2010 at ~ three week intervals until November, 2011 (16 samples points). Needles were collected between 11 am and 1 pm (zeitgeber time [ZT] = 05:00 to 07:00) from 13 individual trees. Environmental data (sunrise; sunset; day length; cumulative weekly precipitation; minimum and maximum daily temperature) was collected over the duration of this experiment (Additional file 2). Sampling intervals and RNA-seq sample sizes for the annual study are summarized in Additional file 1.

Trees used for diurnal analysis derive from the warm/dry region (43.3° N, −123.1° W, 429 m elevation) and were grown in Corvallis, Oregon, USA (42.3° N, −122.9° W, 74 m elevation; Additional file 1). Needles were collected from 6 individual three-year old trees three half-sib families, two sibs per family). Needles were collected at 4 h intervals, starting at 2 AM, for a total of 48 h in early fall (September 7, 8). For this experiment, sunrise (ZT0) occurred at 6:44 AM, and sunset occurred at 19:34 PM, giving a 12:50 photoperiod. Sampling intervals and RNA-seq sample sizes for the diurnal study are summarized in Additional file 1.

### Needle collection, RNA isolation and RNA sequencing

For annual analysis, needles (8–12 total) were collected 2.5–5 cm basipetal of apical buds from four branches (2–3 needles each) representing the four cardinal directions of a single tree. Subsequent samples continued sampling branches from the same cohort of mature needles, instead of new growth. For diurnal analysis, mature needles from the previous season’s growth were similarly collected 2.5–5 cm basipetal of the bud scar, from four branches (2–3 needles/branch). All needles were flash-frozen in liquid N_2_, and ground at dry ice temperatures (FastPrep-24 mill; MP Biomedical, Solon, OH, USA). Ice-cold extraction buffer (1.5 mL of 3 M LiCl/8 M urea; 1% PVP K-60; 0.1 M DTT; [44] was added to ground tissue, homogenized, then centrifuged at 200 *g* × 10 min., 4 °C. The supernatant was incubated overnight at 4 °C, and crude RNA was pelleted (20,000 *g* × 30 min., 4 °C) and cleaned using the ZR Plant RNA MiniPrep kit (Zymo Research, Irvine, CA, USA) and DNase treatment (Turbo DNase; New England Biolabs, Ipswich, MA, USA). RNA concentration was estimated using a Qubit fluorometer (Invitrogen, Carlsbad, CA, USA), and RNA quality was checked using an Agilent BioAnalyzer (Agilent, Santa Clara, CA, USA).

Illumina RNA-seq libraries used 2 μg total RNA and TruSeq chemistry with single indexing adapters (Illumina Inc., San Diego, CA, USA), modified for strand-specific sampling [45]. In this protocol, first-strand synthesis products were desalted to remove unincorporated dNTPs (Sephadex G-25; Sigma-Aldrich, St. Louis, MO, USA), and reconstituted in dNTP-free second-strand synthesis buffer with second strand enzyme mix (New England Biolabs) and a dUTP/dNTP mixture (ThermoScientific, Waltham, MA, USA) to incorporate dUTP into the second strand. All other steps follow Illumina protocols, except that uracil-containing strands were degraded using a uracil-specific excision reagent mixture (37 °C for 15 min; New England Biolabs) prior to PCR. Amplified libraries were quantified and pooled at equimolar 6-plex representation at 10 nM. Single-end 101 bp sequencing was performed at Oregon State University’s Center for Genome Research and Biocomputing (Corvallis, OR, USA) using a HiSeq 2000 (Illumina Inc.) with version 3.0 chemistry and demultiplexing performed using Casava v1.8 (Illumina Inc.). The experiment included 179 libraries sequenced on 34 lanes from 10 different flow cells (GenBank Short Read Archive SRP018395; GEO Accession GSE44058; Additional file 1).

### Transcriptome assemblies and annotation.

Single-end reads from individual trees were combined over all time periods to create 19 single-tree source files. Reads were quality trimmed using Trimmomatic v.0.30 [46] (using options –phred33 LEADING:20 TRAILING:20 SLIDINGWINDOW:5:20), and 19 individual transcriptome assemblies were de novo assembled using *Trinity* v.r2013_08_14 [47] using a minimum size of 200 bp. To create a pan-transcriptome reference, the longest transcripts from each *Trinity* component in single-tree de novo assemblies were identified and combined into a single file, and transcripts smaller than 300 bp were removed. This combined file was sorted by sequence length and then clustered using USEARCH v.7.0.1001 [48]. The usearch64 -cluster_smallmem command was used with a sequence identity threshold of 90% (−id 0.9 flag) and with the -strand both flag to combine forward and reverse transcripts into the same clusters. A table of the number of input bases from each library, transcripts assembled for each individual, and the combined clustered reference assembly is provided in Additional file 1. Individual assemblies and the pan-transcriptome reference are available for download at the TreeGenes Forest Tree Genome database web site under the link “*Pseudotsuga menziesii* Transcriptome” [49].

To annotate plant-derived transcripts, we used BLASTX and TBLASTX [50] to identify transcript matches from the NCBI NR database (minimum identity; expect <1e^−10^), and BLASTX to identify matches to the Mercator plant metabolic function database [51–53]. We used LASTZ [54] to identify chloroplast and mitochondrial transcripts using the *Pseudotsuga sinensis* chloroplast (NC_016064.1) and Loblolly Pine draft mitochondrial [55] genomes as references. We used BLAT [56] to search for conservation between Douglas-fir transcripts and conifer genome references, the Loblolly pine reference genome [57] (*Pinus taeda* version 1.0), and a pre-publication draft for the Douglas-fir genome [58] (version 0.5). LASTZ and BLAT searches used a match criterion of ≥80% identity with contiguous hits ≥50 bp. Annotations are available at the TreeGenes Forest Tree Genome database web site under the link “*Pseudotsuga menziesii* Transcriptome” [49].

### Detecting diurnal and annual cyclic transcriptome variation

RNA-seq reads from individual tree samples were aligned using BowTie 2.2.3 [59] with the following call: bowtie2 --end-to-end -D 15 -R 2 -L 22 -i S,1,1.15. This allowed 15 consecutive seed extension attempts before the aligner moved on (−D 15), a maximum of 2 attempts to re-seed reads with repetitive seeds (−R 2), and a 22 bp seed (−L 22) with zero allowed mismatches in this seed. The function to determine the interval between seed substrings during multi-seed alignment was set to f(x) = 1 + 1.15*sqrt(x), where x is read length (−i S,1,1.15); based on 101 bp read lengths, this resulted in an interval of 13 bp. For this experiment, transcripts showing a median ≤ 5 counts were considered background noise and were excluded from subsequent analyses.

For transcripts exceeding the detection threshold, the 72 diurnal samples (six individuals; 12 time points) were collected into one table and transcript counts were normalized using DESeq [60–62]. Annual samples were similarly tabulated, median filtered, and DESeq normalized. After normalization, we computed family means by averaging reads from half-siblings (trees 44 and 90 = family A; trees 43, 46, and 49 = family B). For the annual study, counts were linearly interpolated to emulate equally-spaced sample intervals. The two processed count tables (diurnal, annual) were passed through JTK-Cycle [41] to identify statistically significant cyclic transcription (*p* ≤ 0.05). The nonparametric test used in JTK-Cycle can identify transcripts as significantly cyclic even if they show minute amplitudes, such as those that might result from circadian fluctuations in total RNA levels [63, 64]. Since the magnitude of daily RNA fluctuation is unknown for conifer needles, we adopted a more stringent false-discovery rate of 1% (*q ≤* 0.01) [65] to identify “high-confidence” cyclic patterns. We used JTK-Cycle to identify the phase (time point at which the underlying curve reaches its maximum value) for each transcript, with phases measured in hours after 12:00 AM for the diurnal study, or Julian days (days after January 1) for the annual study. Summaries of cyclic properties (phase; amplitude; period) are provided in Additional file 3.

### Defining relationship between transcriptional phases and solar and weather factors, and enrichment/depletion tests by season

To evaluate the relationship between the timing of maximum annual gene expression (phase) and environmental variables, annual transcriptional phases were sorted into two week bins, starting on the first sample date (27-October-2010), and continuing until the last sample date. Counts of transcripts reaching maximum expression within two week bins were tallied and evaluated for their association with three environmental variables using ordinary least squares and multiple regression. For this test, the following environmental variables were used as predictors for the timing of maximum transcript accumulation: the mean biweekly high temperature (°C), the sum of biweekly precipitation (in mm), and the mean biweekly photoperiod (in minutes), with photoperiod modeled using a polynomial fit (degree = 2). To reduce correlation among polynomial terms, predictors were mean-centered prior to analysis. Dependent variables were also log_2_ transformed to meet assumptions of normality. The R library gvlma was used to test model assumptions, and the vif function from the R package car was used to estimate variance inflation factors to test for multicollinearity among predictors.

To evaluate Mercator metabolic terms for enrichment or depletion, we binned significantly cyclic transcripts (e.g., *q* ≤ 0.01 from JTK-Cycle) from diurnal and annual experiments into four temporal “phase bins” of equal time duration. For the diurnal experiment, phase bins were six hours in length, and approximately centered on sunrise, solar noon, and sunset: these include “sunrise” (4:01 am – 10:00 am), “midday” (10:01 am – 16:00 pm), “sunset” (16:01 pm – 22:00 pm), and “midnight” (22:01 pm – 04:00 am). Annual bins were 91 or 92 days in length, and approximately centered on the annual solstices and equinoxes; these include “short photoperiod” (5-Nov to 4-Feb), “spring photoperiod” (5-Feb to 5-May), “long photoperiod” (6-May to 5-Aug), and “fall photoperiod” (6-Aug to 4-Nov). Enrichment tests for Mercator pathway terms were performed using term lists for transcripts identified as significantly cyclic and identified to a function (all Mercator bins except 35.2, which is “not assigned.unknown undefined”). Enrichment/depletion tests were performed using a one-tailed Fisher’s exact test and the program Mefisto [66]; adjustments for two-tailed tests were made by multiplying *P*-values by 2, as recommended by Rivals et al. [67] and a false-discovery rate correction of 1% (*q* ≤ 0.01; [65]) was applied using the p.adjust function in the R library stats.

### Comparing annual transcriptome expression variation to other conifers

We compared our annual RNA-seq expression data with results from two previously-published studies examining seasonal transcript variation in conifers. First, Hess and colleagues [33] recently evaluated transcript accumulation in Douglas-fir over a 4 month period (May – September) that corresponds to our “long photoperiod” season. These authors identified six categories of transcripts that show different responses to environmental factors - transcripts that are down-regulated when day length is long (‘day length-down’) and up-regulated when day length is long (‘day length-up’); transcripts down-regulated when temperature is high (‘temperature-down’) and up-regulated when temperature is high (‘temperature-up’); and transcripts down-regulated when total available water is high (‘precipitation-down’) and up-regulated when total available water is high (‘precipitation -up’). Putative orthologous transcripts for these environmentally-responsive transcripts were identified in our pan-transcriptome reference using BLASTN matches. The timing of maximum annual expression (phase) for these genes was determined from our analysis, and counts of transcripts reaching phase in the four annual photoperiod (seasonal) bins defined in this study were tallied into contingency tables. The distribution of counts for all transcripts in common to the two studies were treated as a reference (null) distribution for comparing distributions for each environmentally-responsive category of transcripts. Comparisons and *X* ^*2*^ tests were performed using the CrossTable function in the R library gmodels.

Second, Holliday and colleagues [34] used a microarray-based approach to examine fall needle gene expression in Sitka spruce (*Picea sitchensis*) from British Columbia, Canada over a six week period (August 30 – October 18) that corresponds to our “fall photoperiod” season. Putative orthologues of Douglas-fir and Sitka spruce transcripts were identified using reciprocal best BLASTN matches between the Douglas-fir pan-transcriptome reference and 18,237 clone sequences used in the spruce 21.8 k microarray [34]. We evaluated the direction and magnitude of expression patterns between studies by computing the ratio of fall:summer gene expression using transcripts showing significant expression differences (*q* ≤ 0.05) in microarray data [34]

$$ {ratio}_{microarray}=\left(\frac{\overset{-}{x}\ {intensity}_{18- October}}{\overset{-}{x}\ {intensity}_{30- August}}\right) $$

and transcripts showing significant circannual rhythm (*q* ≤ 0.01. from JTK-cycle) in RNA-seq data.

$$ {ratio}_{RNA-seq}=\left(\frac{\overset{-}{x}\ {counts}_{7- October}}{\overset{-}{x}\ {counts}_{17- August}}\right) $$.

For the 760 transcripts meeting these criteria, expression ratios were ranked from high-to-low and ranks were compared by Kendall’s tau (*τ*) with the cor.test function in R. In this comparison, *τ* is bounded by +1 and −1, with the bounds representing perfect preservation of ranked gene expression ratios in the same (+1) or opposite (−1) direction, and 0 representing random ordering of gene expression ratios between experiments.

## Results

### Defining the needle ‘pan-transcriptome’ of Douglas-fir**–**

**Fig. 1:**
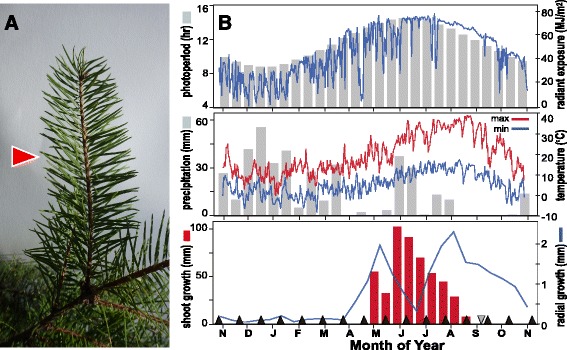
Inputs for the diurnal and annual needle transcriptome study. **a** Douglas-fir branches, showing the location of sampled needles (*red arrow*). **b** Annual environmental conditions and growth rhythm for trees used in this experiment. Shown are: upper panel, light conditions with photoperiod (*bar chart*) and radiant exposure (*blue line*); middle panel, weather conditions with precipitation (*bar chart*) and temperature (*high = red line*; *low = blue line*); lower panel, growth rhythm with terminal shoot growth (*bar chart*) and radial growth (*blue line*). Annual needle sample times are identified in the lower panel using black triangles. The date of the diurnal study is identified as a grey triangle

In this study, needle tissue was sampled by mRNA-Seq to evaluate diurnal and circannual variation in global transcription (Fig. 1a, b; Additional file 1). Needles were sampled for RNA at different time intervals to evaluate two transcriptome responses: (1) diurnal responses, using a sampling interval of four hours across two days (12 time points); and (2) circannual responses, using a sampling interval of approximately 3–4 weeks across a complete year (16 time points). This sampling scheme resulted in a data set that included 19 trees and 179 individual RNA-seq libraries to evaluate different aspects of temporal needle gene expression.

Individual tree mRNA-seq libraries from needles yielded 94.0–573.8 million reads, and individual de novo assemblies using *Trinity* produced 47,976–126,355 components 200 bp or larger (Additional file 1). The number of assembled *Trinity* transcriptome sequences and cumulative sequence length were positively and significantly correlated with the number of input reads (*r* ^*2*^ ≥ 0.92; Additional file 1). Across all assemblies, the majority of *Trinity* components (85.7%) showed a single subsequence, while the maximum number of sequences per component in any library was 227. A ‘pan-transcriptome’ reference was created using the longest sequence from each transcript model in single-tree de novo assemblies, followed by clustering to reduce allelic and redundant sequences to one representative sequence. This step reduced the pool of transcripts from 1.66 million sequences from 19 individual-tree assemblies, to a pan-transcriptome with 199,471 sequences.

Multiple sources of evidence were used to characterize plant-derived transcripts for homologies and putative functions (Additional file 1). BLASTX searches of the plant-specific Mercator plant metabolic database [51, 52] identified homologies for 46,436 transcripts, while BLASTX and TBLASTX searches of NCBI NR database identified tentative identities for 54,384 transcripts. Homology searches against draft conifer genomes identified 102,714 homologs from the Loblolly pine v. 1.0 reference genome, and 167,821 homologs to the Douglas-fir v. 0.5 draft assembly. In total, these searches identified 173,882 transcripts (159.2 Mbp) as derived from Douglas-fir, with 143 originating from the chloroplast genome, 196 from the mitochondrial genome, and 173,544 originating from the nuclear genome. Of the remaining 25,589 transcripts, 19,000 were positively identified by BLAST as derived from foliar metaflora/metafauna present on Douglas-fir needles, or contaminants from the sampling/library construction process. A final list of 6589 transcripts could not be identified using BLAST searches or searches of either draft gymnosperm genome assembly; due to their uncertain origin, these transcripts were omitted from subsequent analyses.

### Fall diurnal transcriptome variation in Douglas-fir tracks daily light/dark transitions

Experiments to detect diurnal cyclic transcriptome variation included six individuals (two sibs per family from three families, sampled in September) collected over twelve 4-h intervals (Additional file 1). After mapping reads from individual diurnal libraries to the Douglas-fir pan-transcriptome reference, 41,382 transcripts met our threshold for analysis of diurnal cycling (median mapped reads >5; Table 1, Additional file 3). Following DESeq count normalization, JTK-Cycle identified 15,487 transcripts as showing significant cyclic diurnal expression at a false-discovery rate of 5% (*q ≤* 0.05), and 12,042 transcripts at a false-discovery rate of *q ≤* 0.01.

**Table 1:** Numbers of transcripts from Douglas-fir showing evidence of diurnal and annual cycling

TRANSCRIPTS	
Total assembled	199,623
Total in genome	173,882
DIURNAL	
Expressed (median > 5 counts)	41,382
Cyclic (*q* ≤ 0.05)	15,487
Cyclic high-confidence (*q* ≤ 0.01)	12,042
ANNUAL	
Expressed (median > 5 counts)	36,145
Cyclic (*q* ≤ 0.05)	24,688
Cyclic high- confidence (*q* ≤ 0.01)	21,225

**Fig. 2:**
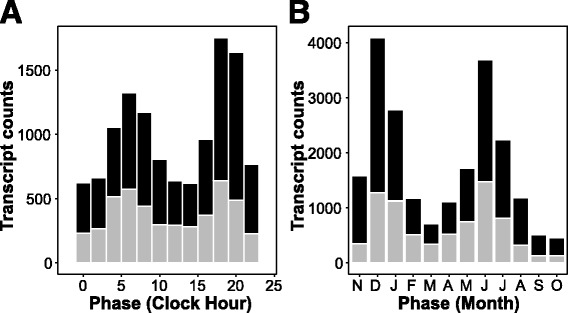
Frequency histograms of estimated phase times for transcripts showing cyclic expression patterns. **a** Histogram of phase for 12,042 transcripts showing significant diurnal cycling. Shown are counts per two hour interval, starting at clock hour 12:00 AM. **b** Histogram of phase for 21,225 transcripts showing significant annual cycling. Shown are transcript counts per one month interval, starting at November 1. In both plots, counts of transcripts with Mercator definitions are shown with black fill, while transcripts lacking Mercator definitions are shown with grey fill

**Fig. 3:**
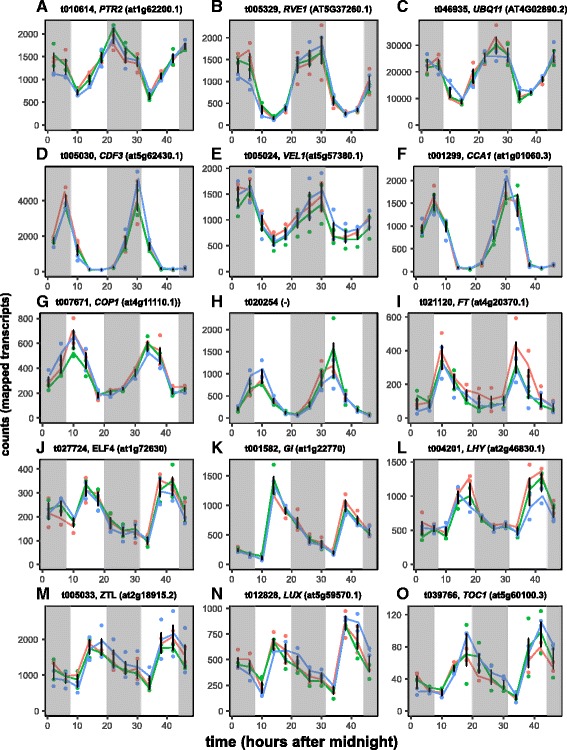
Example expression profiles for transcripts exhibiting significant cyclic diurnal variation, ordered by phase. Gene expression (DESeq-normalized counts per transcript) was estimated by RNA-seq from needle samples representing three families of trees at four hour intervals for two days. For each gene, the Douglas-fir transcript name, putative gene name, and best BLAST match to *Arabidopsis* (prefix ‘at’) are provided. Lines connect the mean expression for each family; error bars represent the SD for all replicates. Absence of shading indicates daylight hours, while shading indicates night hours. Shown are transcripts reaching phase at 0:00 (**a, b**), 2:00 (**c**), 4:00 (**d-f**), 7:00 (**g**), 10:00 (**h**), 12:00 (**i, j**), 18:00 (**k, l**) and 20:00 (**m-o**)

The distribution of expression phase times (Fig. 2a) across all high-confidence diurnal transcripts shows a pronounced bimodal pattern, with the highest proportion of genes reaching maximum transcript accumulation near sunrise (ZT0 = clock hour 6:44 AM) and before sunset (ZT = 12:50, or clock hour 19:34 PM). In total, 5698 (47.3%) of all diurnal transcripts reached phase within +/− two hours of sunrise or sunset. Our high-confidence diurnal transcripts include homologues to many of the known core clock genes from angiosperm models [4, 7] and the distantly related gymnosperm *Cryptomeria japonica* [31] (Fig. 3; Table 2; Additional file 1). We compared the timing of maximum expression for representative clock and seasonal genes in Douglas-fir to values reported for *Arabidopsis* and *Cryptomeria*. Transcripts encoding homologs of *circadian clock-associated1* (*CCA1), cryptochrome1 (CRY1), constitutive photomorphogenic 1 (COP1), vernalization insensitive 3 (VIN3), reveille 1 (RVE1), gigantea (GI), timing of CAB expression 1 (TOC1), lux-arrhythmo (LUX)* and *early flowering 4* (*ELF4.3)* all reached maximum expression within 3 h of the reported maximal expression for one or both of *Arabidopsis* and *Cryptomeria* (Table 2). A smaller number of genes showed pronounced differences in phase relative to *Arabidopsis*. These include *flowering locus T* (*FT*) from Douglas-fir, *late elongated hypocotyl* (*LHY*) from Douglas-fir and *Cryptomeria*, and *Zeitlupe* (*ZTL*) from Douglas-fir and *Cryptomeria.* These differences may be due to incorrect assessments of orthology in gene families, differences in analytical methods, or real differences in the timing of expression in gymnosperm and angiosperm clock genes. A noteworthy finding is that a large proportion of high-confidence diurnal cycling transcripts (38.4%; *N* = 4627) have no homology to known proteins in Mercator and are annotated as “not assigned.unknown” (Fig. 2a). This proportion is similar to reports for diurnal transcripts from the gymnosperm *Cryptomeria japonica*, where 40% could not be identified or classified in BLAST searches [31]. In combination, these studies indicate that many diurnally-cycling transcripts from gymnosperms and angiosperms have diverged to the point that homology cannot easily be assessed. Alternatively, gymnosperms could possess novel clock-dependent components (genes or non-coding RNAs) that lack homologs in angiosperms.

**Table 2:** Diurnal and annual transcriptional phases for core clock genes in Douglas-fir needles, as compared to the angiosperm model *Arabidopsis* and the gymnosperm *Cryptomeria japonica*

Gene Description	Transcript or Locus			Diurnal Phase (clock time)^a^			Annual Phase^b^	
	Douglas-fir	*Arabidopsis*	*Cryptomeria*	Douglas-fir	*Arabidopsis*	*Cryptomeria*	Experiment Day	Month
*RVE1* (myb-like factor)	t005329	AT5G37260	Shoot-057-45	0:00	3:00	4:00	97	Feb
*VIN3* (vernalization insensitive)	t005024	AT5G57380	N/A	4:00	3:00	N/A	255	Jul
*CCA1* (circadian-clock associated)	t001299	AT1G01060	N/A	4:00	6:00	N/A	*n.s.* ^c^	*-*
*COP1* (constitutive photomorphogenic)	t007671	AT4G11110	HI9HAF202BWYA2	7:00	7:00	8:00	85	Jan
*CRY1* (cryptochrome)	t007387	AT4G08920	AB894544	10:00	10:00	4:00	316	Sep
*FT* (flowering locus T)	t021120	AT4G20370	N/A	12:00	0:00	N/A	12	Nov
*ELF4.2* (early flowering 4)	t027724	AT1G72630	N/A	12:00	15:00	N/A	255	Jul
*LHY* (late-elongated hypocotyl)	t004201	AT2G46830	AB894539	18:00	8:00	4:00	*n.s.*	*-*
*GI* (gigantea)	t001582	AT1G22770	AB894538	18:00	20:00	16:00	97	Apr
*ZTL* (Zeitlupe/F-box domain)	t005033	AT2G18915	AB894542	20:00	10:00	16:00	*n.s.*	*-*
*TOC1* (timing of CAB expression)	t039766	AT5G60100	AB894541	20:00	19:00	20:00	*n.s.*	*-*
*LUX* (myb-like transcription factor)	t012828	AT5G59570	N/A	20:00	18:00	N/A	*n.s.*	*-*
*ELF4.3* (early flowering 4)	t029722	AT2G06255	N/A	20:00	20:00	N/A	249	Jul

^a^Douglas-fir diurnal phase values expressed as clock hours, where sunrise occurred at 6:44 AM, and the photoperiod was 12:50 in length. Arabidiopsis thaliana phase estimates were reported as hours from ZT (lights on), based on a 12 h photoperiod [7]; to make units comparable, we added 7 h to *Arabidiopsis* phase values to approximate sunrise (in clock hours) in our study. *Cryptomeria japonica* phase estimates were reported in clock hours [31]

^b^Annual phase values are expressed in experiment days (days after 29-October)

^c^Profiles identified as not significantly rhythmic by JTK_CYCLE are noted “*n.s*”

**Fig. 4:**
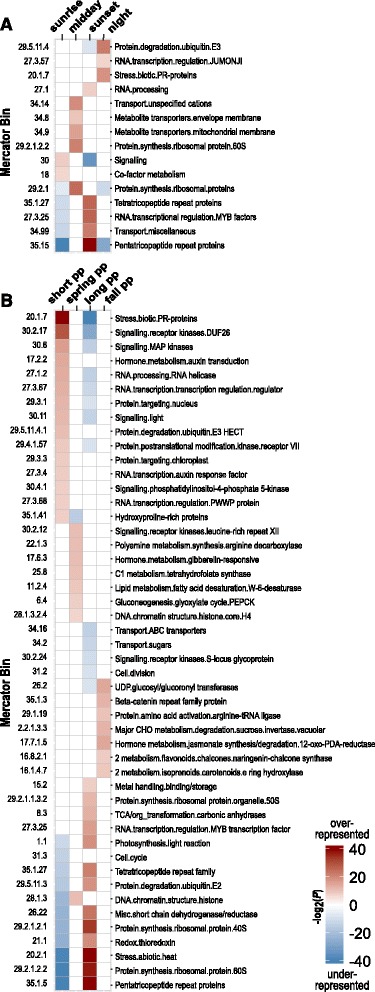
**a** Mercator metabolic categories showing evidence of significant enrichment (*red*) or depletion (*blue*) in the diurnal study. Transcripts were sorted by phase into one of four time bins; ‘sunrise’ (4 – 10 am), ‘midday’ (10 am – 4 pm), ‘sunset’ (4 – 10 pm), and ‘midnight’ (10 pm – 4 am). Shading is scaled by the –log2 value of the *P*-value, following an FDR correction of 0.05. **b** Metabolic pathways showing significant enrichment (*red*) or depletion (*blue*) in four photoperiod categories: short (5-Nov to 4-Feb), spring (5-Feb to 6-May), long (7-May to 5-Aug), and fall (6-Aug to 4-Nov). Shading is scaled by the –log2 value of the *P*-value, following an FDR correction of 0.05

From these 12,042 high-confidence fall diurnal transcripts, we were able to assign Mercator metabolic terms to 7415 transcripts, and analyze these terms for overrepresentation or underrepresentation by categorizing transcriptional phases into bins representing four times of day: ‘sunrise’ (1916 transcripts), ‘midday’ (1333 transcripts), ‘sunset’ (2685 transcripts), and ‘midnight’ (1481 transcripts). Across daily bins, we found evidence for overrepresentation or underrepresentation in 15 Mercator metabolic pathways (Fig. 4a; Additional file 4). Overrepresented terms at sunrise include genes associated with light-responsive signaling (e.g., *phyB, cry1*, glutamate receptor- and cyclic nucleotide-gated ion channels proteins) and enzymes responsible for co-factor biosynthesis (e.g., biotin [holocarboxylase synthetase], thiamin [hydroxyethylthiazole kinase], CoA [phosphopantothenoylcysteine synthetase]). Overrepresented terms at midday include genes associated with protein synthesis (40S and 60S ribosomal proteins), and carbohydrate, nitrate, nucleotide, and small ion transporters. At sunset, overrepresented terms included diverse RNA modifying pathways, including pentatricopeptide repeat gene families (responsible for organelle RNA editing and processing), MYB transcription factors, and RNA processing genes (RNA pol I specific initiation factor *RRN3*; transducing/WD40 repeat proteins; mRNA decapping proteins; methyltransferases). Finally, overrepresented terms at midnight include genes associated with biotic stress (TIR-NBS-LRR proteins; LRR and NB-ARC proteins; *ADR1*-like proteins), *JUMONJI*-like histone demethylases known to play a role in the evening-phase of the *Arabidopsis* circadian clock, and protein degradation pathways based on ubiquitination/de-ubiquitination.

### Annual transcriptome variation in Douglas-fir tracks annual variation in photoperiod

Experiments to detect circannual variation included 5 individuals collected at 16 time points over 12 months, and the data were median filtered, normalized by DESeq, averaged by family, and then linearly interpolated to even sampling dates (Additional file 1). After mapping reads from individual annual libraries to the pan-transcriptome reference, 36,145 transcripts met our threshold for analysis (Table 1). Following DESeq count normalization, JTK-cycle identified 24,688 transcripts showing significant circannual expression at a false-discovery rate of 5% (*q ≤* 0.05). After imposing a more stringent false-discovery correction (*q ≤* 0.01), the list contained 21,225 high-confidence circannual transcripts (Table 1; Additional file 3).

**Fig. 5:**
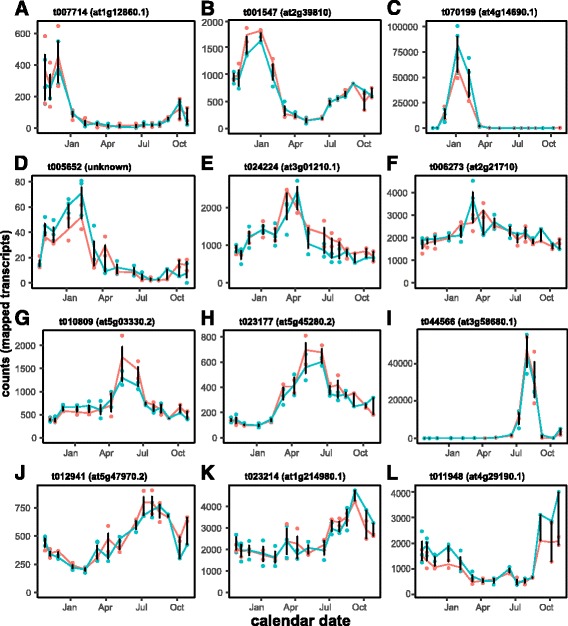
Example expression profiles for transcripts exhibiting significant cyclic annual variation, ordered by phase. Gene expression was estimated by RNA-seq from needle samples collected from two families of trees at *c.* three week intervals for one year. For each transcript, the Douglas-fir transcript name and best BLAST match to *Arabidopsis* (prefix ‘at’) are provided; genes lacking a BLAST match to the NCBI NR database are identified as ‘unknown’. Lines connect the mean gene expression (DESeq-normalized counts per transcript) for each family; error bars represent the SD for all replicates. Shown are transcripts reaching phase in in each experiment month, starting in November (**a**), continuing sequentially through December – September (**b-k**), and ending in October (**l**)

The distribution of circannual expression phases (Fig. 2b) for high-coverage transcripts also shows a pronounced bimodal pattern, with the majority of transcripts reaching maximum expression in one of two seasons: (a) December through January, coinciding with winter dormancy, maximum freeze tolerance, reduced metabolic activity, and shortest photoperiods (day length ≤ 10.5 h); or (b) June through July, coinciding with maximum shoot growth, high metabolic activity, and longest photoperiods (day length ≥ 14.5 h). In total, 10,326 (48.7%) of all circannual transcripts reached phase within +/− 20 days of winter and summer solstices. Example transcripts showing estimated phases for each month of the year are provided in Fig. 5, and they are arranged in increasing order of lag month (e.g., November through October, panels A – L). Details for these transcripts are provided in Additional file 1. Example transcripts reaching peak activity in short photoperiods/winter include transcripts homologous to genes known to play a role in winter adaptation and cold tolerance in *Arabidopsis* (Fig. 5a, inducer of CBP expression, *ICE1*), transcriptional regulation in dormancy (Fig. 5b, mRNA adenosine methylase), and winter photoprotection in conifers (Fig. 5c, early light inducible protein, *ELIP1*; [26]). Example transcripts reaching peak activity in spring photoperiods include transcripts with unknown functions (Fig. 5d), oxidoreductase and RNA-binding activity (Fig. 5e), and mitochondrial transcription termination factors implicated in adaptation to cold climates in conifers [68] (Fig. 5f). Example transcripts reaching peak activity in long photoperiods/summer include transcripts for a cysteine proteinase (Fig. 5g) and a pectin acetylesterase (Fig. 5h) associated with drought responses in *Arabidopsis* [69], and a multiprotein bridging factor that responds to biotic and abiotic stresses, including heat (Fig. 5i). Example transcripts reaching peak activity in fall photoperiods include transcripts for an FMN-linked oxidoreductase (Fig. 5j) implicated in convergent adaptation to cold climates [68], a chloroplast co-chaperonin (Fig. 5k) associated with drought in *Arabidopsis* [69], and a CCCH-type Zn-finger protein that is upregulated in October (Fig. 5l). As we observed for diurnal transcripts, 36.3% (*N* = 7711) of the high-confidence annual cycling genes are “not assigned.unknown” in Mercator (Fig. 2b), and they show no homology to proteins or RNAs in GenBank.

Mercator terms were associated with 13,514 of the 21,225 high-confidence transcripts (excluding “not assigned.unknown”), and these were analyzed for evidence of Mercator term enrichment or depletion. Transcriptional phases were divided into seasonal bins by photoperiod (defined in Methods); bins included ‘short’ photoperiod (5796 transcripts), ‘spring’ photoperiod (1466 transcripts); ‘long’ photoperiod (5073 transcripts); and ‘fall’ photoperiod (1179 transcripts). Across seasonal bins, we found evidence for over- or under-representation in 48 Mercator metabolic pathways (Fig. 4b; Additional file 5). Pathways showing an over-representation of phase values in short photoperiod days (winter) were related primarily to biotic stress, signaling, protein degradation and post-translational modification, and RNA transcription in diverse regulatory genes such as MAP kinases, RNA helicases, and proteins playing a role in hormone signaling and transduction (e.g., auxin; DUF26). Pathways showing over-representation of phases on long photoperiod days (summer) were related primarily to organelle regulation (pentatricopeptide and tetratricopeptide repeat protein families), photosynthesis-related metabolism (photosynthesis light reaction; carbonic anhydrase; metal binding/storage), ribosomal protein synthesis (nuclear and organellar), redox/thioredoxin, and stress from heat.

We used multiple regression analysis to test whether three environmental factors – two week interval mean photoperiod (in minutes, as second-order polynomial), two week interval mean temperature (maximum °C), and two week interval cumulative precipitation (in mm) – could predict the number of transcripts reaching phase over two week periods across a complete year. Tests for multicollinearity indicated a low to modest level of multicollinearity among the environmental factors (variance inflation factor ≤ 2.82 for all factors; Table 3). The results of the regression model indicated the combined predictors explained 83.41% of the total variance (adjusted *R2* = 0.802, *F*_*4,21*_ = 26.38, *p* = 6.31e-08; Table 3), with mean photoperiod (*β* = 8.468e-01, *p* = 1.25e-07; Table 3) and mean maximum temperature (*β* = −3.739e-01, *p* = 0.0206; Table 3) indicated as the most significant predictors. Mean cumulative precipitation variable accounted for insignificant levels of variation (*β* = −9.424e-03, *p* = 0.9435; Table 3). Photoperiod, temperature, and precipitation have all been implicated as major drivers in seasonal gene expression in different plant tissues [12, 34], but at the scale of global transcription over a year, photoperiod is the dominant driver of the timing of cyclic gene expression maxima in our study of Douglas-fir needles.

**Table 3:** Model summary for multiple regression analysis of relationship between the number of transcripts reaching maximum expression (phase) and environmental predictors (photoperiod, maximum temperature, precipitation) over two week intervals for a full year

ANOVA	df	Sum of squares		Mean of squares	*F*	Pr(>|t|)	
photoperiod	2	27.03		13.51	49.29	1.168e-08	
temperature	1	1.897		1.897	6.920	0.0156	
precipitation	1	0.0014		0.0014	0.0051	0.9435	
residuals	21	5.757		0.2741	*NA*	*NA*	
Coefficients	VIF	b	Std. Err.	β	Std. Err.	T value	Pr(>|t|)
constant	*NA*	-1.307e + 00	1.967e-01	-1.130e-16	8.718e-02	−6.646	1.40e-06
photoperiod	2.531	2.667e-03	1.281e-03	2.945e-01	1.414e-01	2.082	0.0497
photoperiod^2^	1.494	8.037e-05	1.031e-05	8.468e-01	1.087e-01	7.793	1.25e-07
temperature	2.822	−4.886e-02	1.951e-02	−3.739e-01	1.494e-01	−2.503	0.0206
precipitation	2.186	−9.627e-03	1.343e-01	−9.424e-03	1.315e-01	−0.072	0.9435

R2 = 0.834; Adjusted R2 = 0.802; Residual standard error = 0.4445 (21 d.f.); *F*-statistic: 26.38 on 4 and 21 d.f., *p*-value = 6.31e-08

### Generalizing annual transcriptome expression variation to other conifers

To explore the generality of our circannual predictions, we evaluated the timing and direction of annual transcript accumulation (phase) from the perspective of two previously published studies of seasonal gene expression in conifers: summer transcription in Douglas-fir, and fall transcription in Sitka spruce. The recent study by Hess et al. [33] identified six categories of ‘environmentally-responsive’ transcripts, and we used reciprocal BLAST searches of both reference transcriptomes to identify 2278 transcripts common to both studies. These include 537 transcripts that are down-regulated when day length is long (‘day length-down’), 332 transcripts up-regulated when day length is long (‘day length-up’), 363 transcripts down-regulated when temperature is high (‘temperature-down’), 295 transcripts up-regulated when temperature is high (‘temperature-up’), 271 transcripts down-regulated when total available water is high (‘precipitation-down’), and 480 transcripts up-regulated when total available water is high (‘precipitation-up’) (Additional file 1).

**Fig. 6:**
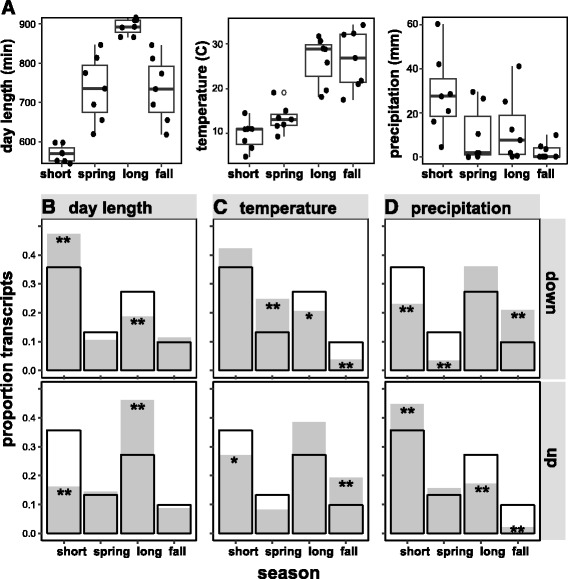
Seasonal distributions of phase times for environmentally-responsive transcripts identified in Hess et al. [33]. **a** Box plots show the mean and interquartile range for mean day length, mean maximum temperature, and cumulative precipitation for two-week intervals at our study site. These three environmental factors are predicted to influence transcription in Douglas-fir. **b**-**d**) Seasonal distributions of predicted and observed numbers of transcripts reaching maximum expression for each of the six environmentally-responsive classes identified in [33]. The predicted distribution (*unfilled bars*) represents the proportion of transcripts for each season reaching maximum expression (phase), across all environmentally responsive transcripts that show significant circannual cycles (*N* = 2278 transcripts). Observed distributions (filled bars) represent the proportion of transcripts for each season reaching maximum expression for each category (e.g., ‘day length-down’). Categories showing a significant excess or deficit of transcripts relative to expected proportions are identified by one or two asterisks (* = *p* ≤ 0.05; ** = *p* ≤ 0.01)

The distribution of phase dates for each of the six environmentally-responsive transcript categories was significantly different than the expected distribution for all ‘environmentally-responsive’ transcripts (minimum *X* ^*2*^ = 36.041, 3 d.f., *p* ≤ 7.34e-8; Additional file 1). In nearly all cases, the distribution of transcript phases (relative to expected distribution; unfilled bars in Fig. 6b-d) are consistent with their predicted environmentally-responsive transcript category (filled bars, Fig. 6b-d) [33], and the seasonal factors that are predicted to drive their expression (Fig. 6a). For example, ‘day length-down’ transcripts are predicted to be down-regulated when day length is long. Our results show that there is a significant excess in the number of transcripts reaching phase during the short photoperiod season (Fig. 6b, upper panel), and a corresponding deficiency in the number of transcripts reaching phase during the long photoperiod season. This trend is reversed for ‘day length-up’ transcripts (Fig. 6b, lower panel); these are predicted to be up-regulated when photoperiod is long, and our results show that there is a significant excess of transcripts reaching phase during the long photoperiod season, and a deficiency in transcripts reaching phase during the short photoperiod season. Similar trends are observed for ‘temperature-responsive’ transcripts, as ‘temperature-down’ show a significant excess of transcripts reaching phase in coldest months (short photoperiod; Fig. 6c, upper panel), and ‘temperature-up’ show a significant excess of transcripts reaching phase in warm months (fall photoperiod; Fig. 6c, lower panel). ‘Precipitation-responsive’ transcripts show a similar pattern, although in this case the ‘precipitation-down’ category shows a significant excess of transcripts reaching phase in the driest months (fall photoperiod season; Fig. 6d, upper panel), and ‘precipitation-up’ shows a significant excess of transcripts reaching phase in the wettest months (short photoperiod).

An important finding in our study is that a significant proportion of transcripts reach peak accumulation during the dormant period (e.g., Fig. 2). If this is a common feature of conifer needle transcription, similar patterns of up- and down-regulation should be observable in other late season time-course studies of gene expression in conifers. To evaluate the reproducibility of this pattern, we selected a subset of dates from our annual study (17-Aug-2011 and 7-Oct-2011) to compare with a microarray-based study of needle gene expression in Sitka spruce (*Picea sitchensis*) conducted at a similar time interval (30-Aug, 18-Oct) in British Columbia, Canada [34]. From these studies, we identified 11,582 transcripts that showed reciprocal best BLASTN matches between the Douglas-fir transcriptome and Sitka spruce clones used in microarray design (Additional file 6). Out of this total, 760 transcripts showed evidence of significant seasonal change in Sitka Spruce (*q* ≤ 0.05; two-fold change [34]), and cyclic behavior in Douglas-fir (*q* ≤ 0.01, JTK-cycle). The rank order of fall:late summer gene expression ratios for these 760 transcripts was more preserved than would be expected if gene order was random (Kendall’s *τ* = 0.2901; z = 11.989; *p* < 2.2e − 16), indicating that the direction of transcript change (increasing, decreasing) and the relative magnitude of transcript accumulation was more preserved than expected across these dates. Due to their differences, comparisons between microarray and RNA-seq data are challenging within designed studies; for this reason, the similarity between fall transcriptional responses in *Picea* vs. *Pseudotsuga* should be considered provisional evidence for a shared response to the onset of fall and short photoperiods, one that is characterized by a significant increase in transcript accumulation prior to the onset of dormancy.

## Discussion

Perennial, evergreen needles are one of the key features that distinguish nearly 700 species of conifers from the tree model *Populus* and from other deciduous angiosperm trees. Over a calendar year, persistent leaves undergo transcriptional and metabolic shifts that allow for photosynthesis when conditions are favorable (even during winter), while providing conservative protection from the damaging effects of winter cold during dormancy [23, 70], and drought during seasonal dry periods [21, 22]. By defining the complexity and contribution of gene expression over the complete growth-dormancy cycle, circannual studies like this provide a foundation for identifying associations between the synchrony of transcriptional change to seasonal events in climate or phenology, and they offer a source of evidence for identifying genes/pathways that may contribute to adaptive responses in forest trees to climate change.

Our results show that the timing of maximum transcript accumulation in diurnally and circanually cyclic transcripts from conifer needles is associated with the timing and amount of light at both temporal scales. At a daily scale, 12,042, or 29%, of expressed diurnal transcripts showed a significant diurnal cycle, with nearly one half of these diurnal transcripts achieving maximum mRNA accumulation within +/− 2 h of sunrise or sunset. This pattern has been shown in diverse plants and animals [5, 7], and is explained as “expression anticipation” for the transition from dark to light in the morning, and light to dark in the evening. We found a high degree of concordance in the phase for these genes in the conifers Douglas-fir and *Cryptomeria* (Table 2) and the angiosperm *Arabidopsis*, which is striking given the different methodologies used in these studies (e.g., microarrays vs. RNA-seq) and the organismal divergence included in the comparison. A small number of genes – specifically, *FT*, *LHY*, and *ZTL* – showed pronounced differences in phase between these three taxa. These differences may be due to incorrect assessments of orthology in gene families, but in the case of *FT and LHY,* it may be due to divergent gene functions in gymnosperms and angiosperms, as has been previously suggested [14, 28]. The influence of light as an entraining force of circadian cycles is well-known in plants [1–4, 7, 8], and our analysis expands the list of known circadian genes to a new lineage of gymnosperms. It’s important to note that our circadian study was performed near the autumnal equinox (September 7–8), so it could represent a different transcript profile than those represented in *Cryptomeria* [31], which were sampled closer to the summer and winter solstices (July 30–31; December 22–23). Given the strong circannual variation observed in Douglas-fir (e.g., Fig. 2) and observations of circadian dampening or arrest during winter dormancy in *Cryptomeria* [31] and other organisms [71], ‘diurnal’ transcriptomes are likely to exhibit different cyclic behaviors and members under different seasonal conditions and tissue sources. Expanding these comparisons to additional tissues, seasons, and conifer species under standardized conditions (age of tissue and plant; sampling interval; analytical methods) with comparable gene expression detection methods (e.g., RNA-seq) would make it possible to identify essential circadian components in gymnosperm genomes, as well as developmentally- or temporally-unique components.

At an annual scale, 21,225, or 58.7%, of expressed annual transcripts showed significant circannual cycles, with nearly half of all expressed transcripts achieving maximum mRNA accumulation within +/− 20 days of the shortest or longest photoperiod. Photoperiod is known to play a crucial role in the timing of the onset of dormancy, bud break and flowering in photoperiod-sensitive plants [11, 14, 17, 72, 73], and photoperiod has been shown to explain greater seasonal variation in photosynthetic activity than temperature for many tree species [74]. Given the reliability of light as an entraining force for forecasting environmental change, photoperiod is a common signal used by many trees for tracking seasons at the molecular level [14, 72, 73], through interactions with phytochromes, the *constans* and *FT* (or *FT*-like) module [11, 14, 75], and regulatory proteins that control circadian expression and bud dormancy and release (e.g., *apetala2*/*EBB1* [12]). Douglas-fir has been characterized as exhibiting ‘photoperiod-sensitive’ and ‘photoperiod insensitive’ responses (summarized in [73]), and our study of Douglas-fir annual transcription shows a strong ‘photoperiod effect’, such that photoperiod is the single strongest predictor of the number of genes reaching peak expression throughout the year (Table 3). At present, it’s not possible to determine the extent to which this global circannual expression pattern is driven by photoperiod, climate, or the photoperiod-sensitive nature of the species. As with circadian studies, expanding circannual studies to photoperiod-sensitive and insensitive conifer species in diverse environments could help to identify the essential and variable components of a ‘core’ circannual clock.

Environmental cues like temperature and water availability are known to modulate seasonal processes and gene expression [73]; in our study, temperature was a significant yet weak predictor of the global number of genes reaching phase (Table 3), and precipitation showed an insignificant relationship to genes reaching phase. Genes responding to these climatic factors may be less abundant in plant transcriptomes, their activity may be temporally transient, or they may be difficult to model using cyclic (cosine-like) functions. Population sampling strategies like those used by Hess et al. [33] may show the greatest power for identifying genes that respond to secondary signals like temperature or water availability. Interestingly, estimates of annual phase from our study were generally congruent with classifications for environmentally-responsive transcripts by Hess et al. [33] (Fig. 6), as transcripts described as upregulated during long photoperiods, high temperatures, and dry conditions were enriched in our ‘long photoperiod’ (also hottest and driest; Fig. 6a) season. Similarly, transcripts described as downregulated during long photoperiods, high temperatures, and dry conditions were enriched in our ‘short photoperiod’ (also coolest and wettest; Fig. 6a) seasons. Examples of transcripts meeting these predictions are shown in Fig. 5a (predicted ‘day length-down’; [33]) and Fig. 5i (predicted ‘temperature-up’; [33]).

While there is general agreement in the predicted expression patterns in these studies, it’s important to note that a sizeable proportion of the ‘environmentally-responsive’ Douglas-fir transcriptome described by Hess et al. [33] reaches maximum activity in seasons that are contradictory to predictions. For example, 289 of the 537 (53.8%) “day length – down” transcripts reach maximum expression during the expected short photoperiod season, but 114 (21.2%) reach maximum expression in the *unexpected* long photoperiod season. Across all six categories described by Hess et al. [33], we find that an average of 19.2% of transcripts reach phase in the opposing season predicted by their environmentally-responsive categories. These discrepancies could be due to analytical errors, genotype x environment interactions imposed by different garden climates, or complex transcription responses to seasonally-recurring stresses.

The entrainment of diurnal and circannual gene expression by light quality or day length in Douglas-fir is intuitive, but the dramatic accumulation of transcripts at the winter solstice presents something of a paradox; *why does transcript accumulation peak for such a high proportion of the cyclic transcriptome during dormancy, when metabolic activity is reduced and growth is arrested?* In western Oregon, Douglas-fir typically remains photosynthetically active during the winter [22], but seasonal transcription for protein synthesis, metabolism, and photosynthesis are underrepresented (Fig. 4). Instead, Douglas-fir undergoes physiological changes that result in maximum cold hardiness between November to early December [25], and this timing coincides with the increase in genes achieving maximum transcript accumulation (e.g., Fig. 2b), and enrichment for genes involved in stress responses, hormone transduction, and light signaling (Fig. 4). Transcription for many genes and pathways are known to increase under short days and cold temperatures in response to adverse environmental conditions [24, 26], such as temperature, osmotic, drought, and light stress. The list of Douglas-fir cyclic transcripts reaching maximum transcription in winter includes genes implicated in cold acclimation of *Populus* buds (e.g., early light-inducible proteins (ELIPs), C-repeat binding factors, fatty acid desaturases, major carbohydrate enzymes, LEA-like proteins, and heat shock proteins [18]). Short day- and dormancy-induced transcriptional upregulation in needles appears extensive, and further effort is required to determine how this seasonally-diverse transcript pool coordinates the proximal demands of growth cessation, preparation for winter stress, and establishment/maintenance of dormancy and cold hardiness.

## Conclusions

Conifers possess evergreen needles that sense and respond to year-round environmental signals. In this study, we used RNA-seq to monitor transcriptional activity in Douglas-fir needles at daily and annual cycles, and we found that gene expression is dependent on light at both scales. At a daily scale, we identified 12,042 transcripts that showed significant cyclic variation, with nearly half of transcripts achieving maximum activity +/− 2 h from sunrise or sunset. At an annual scale, 21,225 transcripts showed significant cyclic variation, with nearly half of transcripts achieving maximum activity +/− 20 days from the winter or summer solstices. Comparisons with diurnal and seasonal gene expression studies with other conifers show a high degree of concordance, suggesting that results from this study may be useful for predicting the timing of transcription in other populations of Douglas-fir, and other species of genomically-complex temperate zone gymnosperms such as spruce [76, 77] and pine [57]. To aid in these comparisons, we have made the daily and circannual transcriptional patterns for Douglas-fir available for examination [78], and have merged our estimates of predicted phase (circadian; circannual) with results from the related study of Hess et al. [33] to provide a comprehensive list of temporally- and environmentally-responsive transcripts (Additional file 7).

The combination of large genome size (~20 Gbp), high transcriptional complexity [79, 80], genic redundancy, and divergence from angiosperm models has made it difficult to infer gene function in conifers based on homology alone; for this reason, genetic-environmental and genetic-phenotypic associations are being investigated in many conifer species [68, 81]. Conifers lack tractable models for reverse genetic manipulation, so context-specific evidence offered by diurnal, seasonal, and circannual gene expression studies provide the insights into the functional relevance of transcript accumulation, and the seasonal context that genes and pathways are up- and downregulated. A clearer understanding of role that complex circannual transcription plays in physiological and fitness responses will emerge as the developing Douglas-fir genome [82] and other conifer genomes are integrated with daily, seasonal, circannual, and tissue-specific transcriptomic studies.

## Additional files

Additional file 1: Additional information on needle sampling methods, original source locations for trees, the location of common gardens, sampling intervals used for collections, individual tree sequencing library summaries, and individual tree transcriptome summaries. The file can be opened in Microsoft Word or any word processing program that accepts rich text format. (DOCX 812 kb)

Additional file 2: Daily environmental data associated with experiment. This file includes date (mm/dd/yyyy), experiment day, Julian day, experiment week, experiment month, minimum air temperature (°C), maximum air temperature (°C), mean daily air temperature (°C), daily precipitation (mm), day length (h:m:s), and the daily sum of solar radiant exposure (MJ/m^2^). The file can be opened in Microsoft EXCEL or any program that accepts text as tab-separated values. (TSV 20 kb)

Additional file 3: Summary of JTK-Cycle inferred cycle properties for diurnal and annual cyclic transcripts. The file can be opened in Microsoft EXCEL or any program that accepts text as tab-separated values. (TSV 3802 kb)

Additional file 4: Summary of enriched and depleted Mercator functional category terms for the diurnal study. The file can be opened in Microsoft EXCEL. (XLSX 1514 kb)

Additional file 5: Summary of enriched and depleted Mercator functional category terms for the annual study. The file can be opened in Microsoft EXCEL. (XLSX 2602 kb)

Additional file 6: Summary of transcripts showing best reciprocal BLAST match between Douglas-fir and Sitka spruce clones used in Holliday et al., 2008, and data used for comparing ranked lists for Kendall’s Tau test. The file can be opened in Microsoft EXCEL. (XLSX 335 kb)

Additional file 7: Summary of transcripts showing best reciprocal BLAST match between Douglas-fir transcripts in Hess et al. 2016 and Douglas-fir transcripts in this paper. The file can be opened in Microsoft EXCEL. (CSV 446 kb)

## Abbreviations

CCA1: Circadian clock-associated
CoA: Coenzyme A
COP: Constitutive photomorphogenic
CRY: Cryptochrome
DTT: Dithiothreitol
ELF: Early flowering
ELIP: Early light inducible protein
FT: Flowering locus T
GI: Gigantea
LHY: Late elongated hypocotyl
LRR: Leucine-rich repeat
LUX: Lux-arrhythmo
MYA: Millions of years ago
NBS: Nucleotide binding site
PHY: Phytochrome
PVP: Polyvinylpyrrolidone
RVE: Reveille
TOC: Timing of CAB expression
VIN: Vernalization insensitive
ZT: Zeitgeber time
ZTL: Zeitlupe
