# Supplementary material for: Transcription through the eye of a needle: daily and annual cyclic gene expression variation in Douglas-fir needles
Source: BMC Genomics. 2017 Jul 24;18:558. doi: 10.1186/s12864-017-3916-y (PMC5525293; doi:10.1186/s12864-017-3916-y)
Supplement: Supplementary file 1 — Additional information on needle sampling methods, original source locations for trees, the location of common gardens, sampling intervals used for collections, individual tree sequencing library summaries, and individual tree transcriptome summaries. The file can be opened in Microsoft Word or any word processing program that accepts rich text format. (DOCX 812 kb) [file 12864_2017_3916_MOESM1_ESM.docx]

## *BMC Genomics* Supporting Information

Article title: **Transcription though the eye of the needle: daily and annual cycles of gene expression variation in Douglas-fir needles**

Authors: Richard Cronn^1^, Peter Dolan^2^, Sanjuro Jogdeo^3^, Jill Wegrzyn^4^, David B. Neale^5^, J. Bradley St. Clair^1^, and Dee Denver^6^

**The following Supporting Information is available for this Article:**

**Additional File 1.** (this document). Additional information on original source locations for trees, the location of common gardens, sampling intervals used for collections, individual tree sequencing library summaries, and individual tree transcriptome summaries. The file can be opened in Microsoft Word or any word processing program that accepts rich text format (RTF).

**Additional File 2.** Daily environmental data associated with experiment. This file includes date (mm/dd/yyyy), experiment day, Julian day, experiment week, experiment month, minimum air temperature (°C), maximum air temperature (°C), mean daily air temperature (°C), daily precipitation (mm), day length (h:m:s), and the sum of radiant exposure (megajoules/m^2^). The file can be opened in Microsoft EXCEL or any program that accepts text as tab-separated values (TSV).

**Additional File 3.** Summary of JTK-CYCLE inferred cycle properties for diurnal and annual cyclic transcripts. This file includes the following variables: TRANSCRIPT (name), DIUR_FLAG (identifies transcripts showing significant diurnal cycle), ANNU_FLAG (identifies transcripts showing significant annual cycle), DIUR_ANNU_FLAG (identifies transcripts showing significant diurnal and annual cycles), DIUR_BH.Q (significance from JTK_ CYCLE, corrected for multiple tests), DIUR_ADJ.P (significance from JTK_CYCLE), DIUR_PERIOD (JTK_CYCLE inferred period length, in hours), DIUR_PHASE (JTK_CYCLE inferred phase for inferred period, in hours from 2:00 a.m. [start of experiment]), DIUR_ PHASE 24 (JTK_CYCLE inferred phase for 24 hour clock, in hours from midnight), DIUR_AMP (JTK_CYCLE inferred amplitude, in mapped RNA-seq reads), DIUR_MEDIAN (median number of mapped RNA-seq reads), ANNU_BH.Q (significance from JTK_ CYCLE, corrected for multiple tests), ANNU_ADJ.P (significance from JTK_ CYCLE), ANNU_PERIOD (JTK_CYCLE inferred period length, in days), ANNUAL_ PHASE (JTK_CYCLE inferred phase for inferred period, in days from experiment start), ANNUAL_ PHASE 365.Julian (JTK_CYCLE inferred phase for 365 day period, in Julian days), ANNUAL_AMP (JTK_CYCLE inferred amplitude, in mapped RNA-seq reads), ANNUAL_MEDIAN (median number of mapped RNA-seq reads), and ANNU_ PHASE.MONTH (JTK_CYCLE inferred phase, expressed as calendar month). The file can be opened in Microsoft EXCEL or any program that accepts text as tab-separated values (TSV).

**Additional File 4.** Summary of enriched and depleted MapMan/Mercator functional category terms for the diurnal study. The file can be opened in Microsoft EXCEL (XLS).

**Additional File 5.** Summary of enriched and depleted MapMan/Mercator functional category terms for the annual study. The file can be opened in Microsoft EXCEL (XLS).

**Additional File 6.** Summary of transcripts showing best reciprocal BLAST match between Douglas-fir and Sitka spruce clones used in Holliday et al., 2008, and data used for comparing ranked lists for Kendall’s Tau test. The file can be opened in Microsoft Excel (XLS).

**Supplemental Notes S1**

**Fig. S1: Location of tree sources and common gardens**. *Upper map –* Douglas-fir seed sources derive from three environments; warm/dry (Oregon Siskiyou Range, low elevation; orange circles), cool/wet (Washington Coast Range, low elevation; green circles) and cold/wet (Washington Cascade Range, high elevation; blue circles). The location of the annual common garden is noted by a black square, and the location of the diurnal garden is noted by a red square. *Lower diagram -* Samples derive from three geographic and climatic zones, with each zone represented by four to five trees (two families per region; two or three samples per family). Samples from all sites were used for pan-transcriptome assembly; only trees from Oregon Siskiyou Range were analyzed for diurnal and annual expression.

**
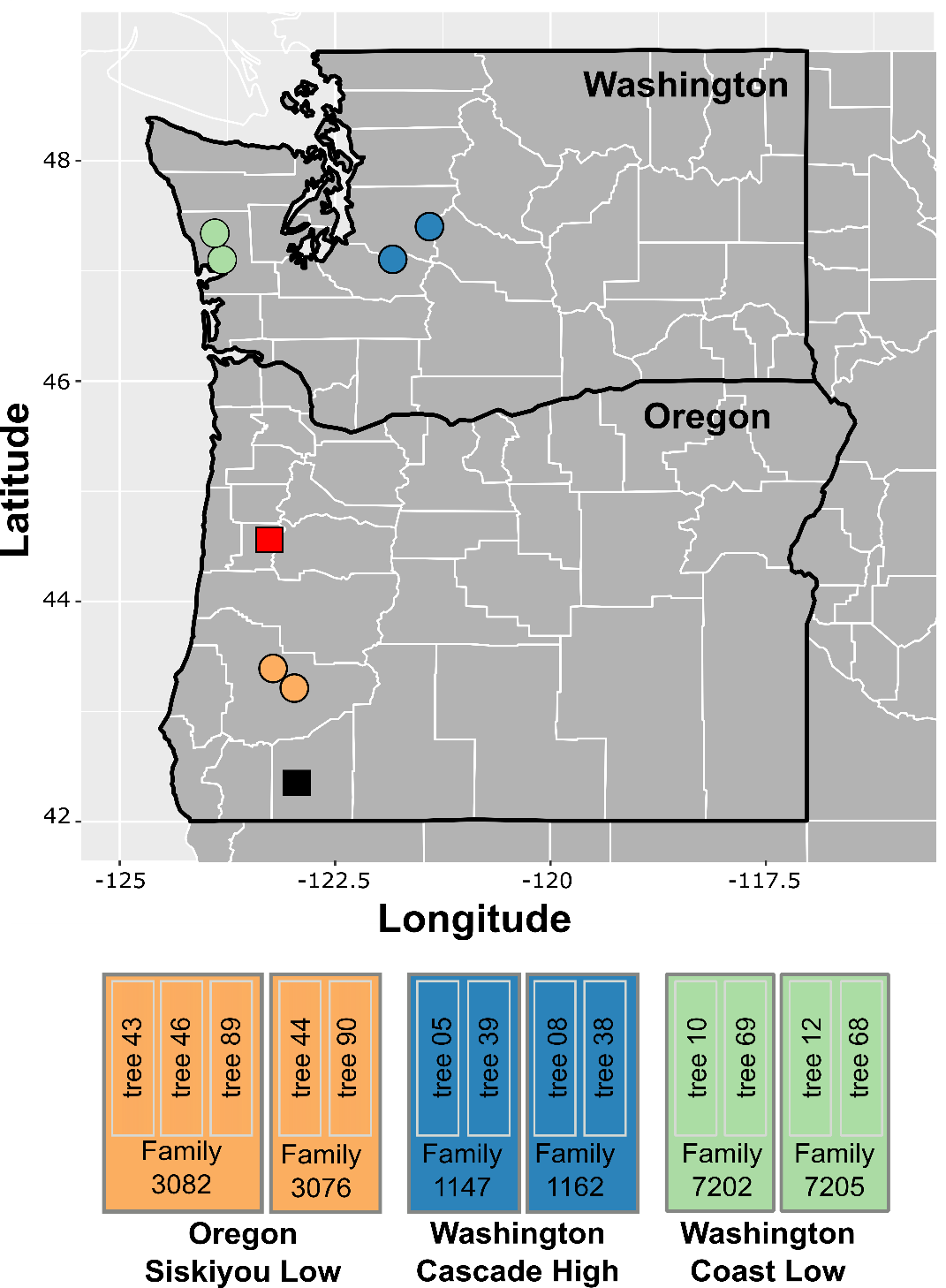
**

**Fig. S2: Annotation of the Douglas-fir needle pan-transcriptome reference.** (A) Transcripts from 19 individual tree assemblies were clustered to 199,623 unique transcripts, and sequences were annotated using similarity matches to four database: the MapMan/Mercator plant metabolism database (Lohse, *et al.*, 2014), NCBI NR database, the draft Loblolly pine genome (version 1.0) and the draft Douglas-fir genome (version 0.5). A total of 173,882 transcripts were identifiable as derived from Douglas-fir in one or more of the searches, with the highest proportions identifiable in conifer genomes. The remaining 25,741 transcripts (not shown) failed to match plant genomes, and include foliar metaflora/fauna or contaminants (not tree) and unknown sequences. (B) Distribution of transcript annotations based on MapMan/Mercator bins.


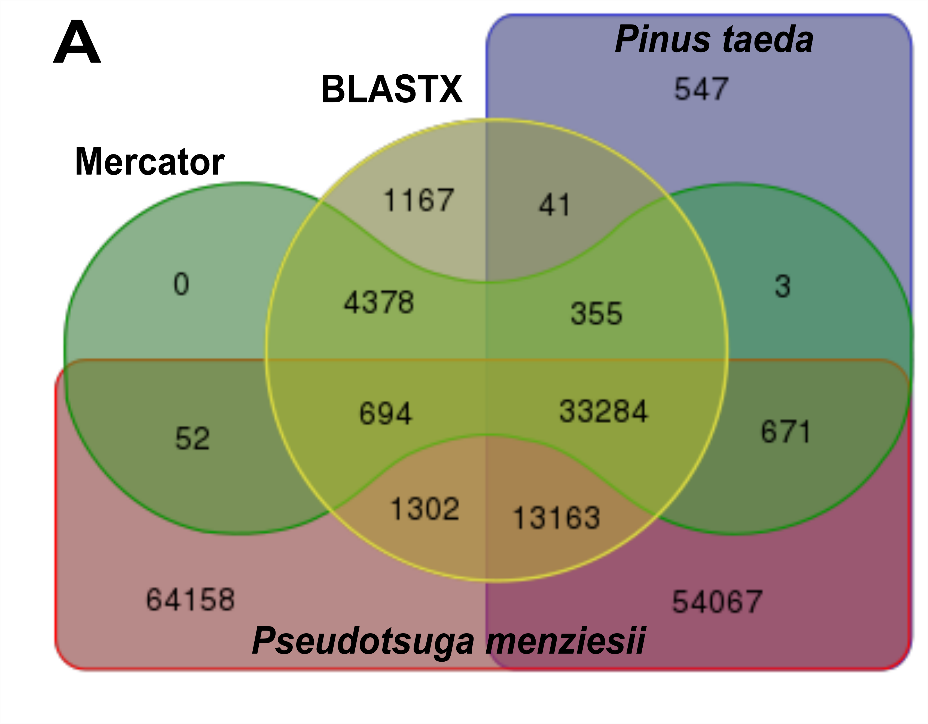


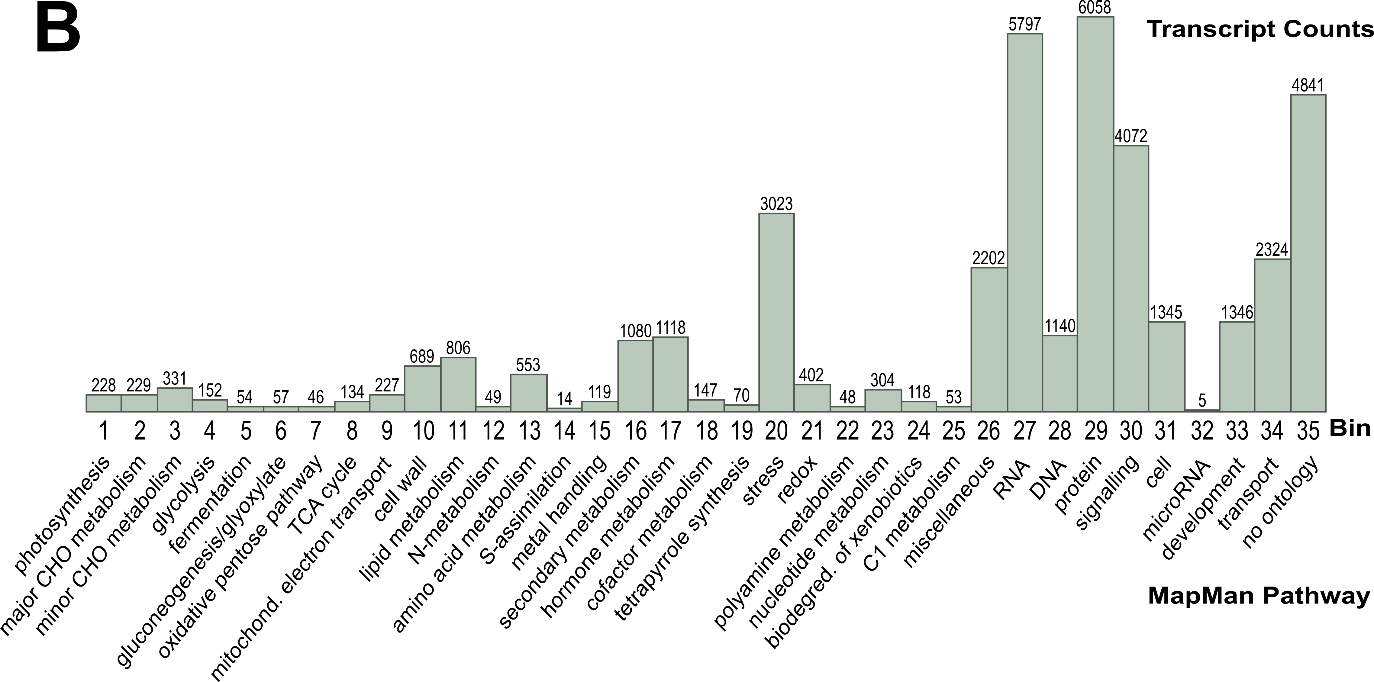


**Fig S3:** Relationship between individual sequence inputs and resulting individual Trinity transcriptome assemblies. We observed a significant positive relationship between the number of input trimmed reads (or input bases) and the resulting number of Trinity components (transcripts), total Trinity sequences (components and subcomponents), and the cumulative transcriptome assembly size. We observed a significant inverse relationship between sequence inputs (trimmed reads; input bases) and the transcriptome contig N50 size.

**
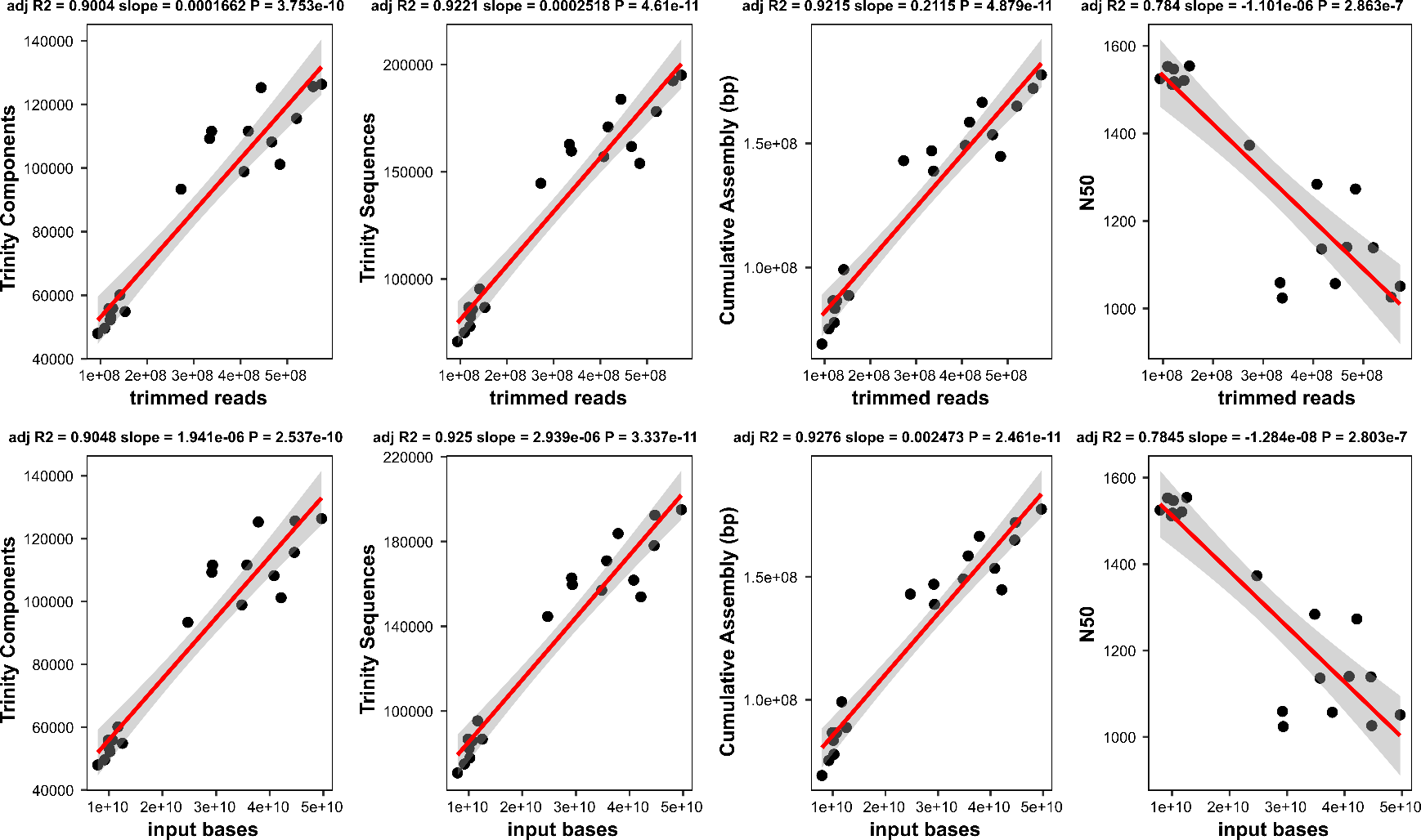
**

**Table S1:** Sampling intervals and Douglas-fir needles samples collected for transcriptome assembly and the annual cycling study. Needles were collected at approximately three week intervals, starting at October 27. For this experiment, transcriptome samples from single-tree needle RNA-Seq libraries were used to construct individual tree de novo references. Once the reference was constructed, reads from Oregon Siskiyou range (OR_SIS) trees were mapped back to determine counts per sampling interval for each tree; trees from Washington (WA_CAS, WA_CST) were only used for reference construction, and are not analyzed for cyclic expression in this study. The number of reads (in millions) obtained per sample are indicated. Missing samples (due to insufficient mRNA) are noted as “-”

| Source | Family/  Tree | 27-Oct-2010 | 9-Nov-2010 | 30-Nov-2010 | 6-Jan-2011 | 7-Feb-2011 | 23-Feb-2011 | 10-Mar-2011 | 7-Apr-2011 | 6-May-2011 | 17-Jun-2011 | 7-Jul-2011 | 28-Jul-2011 | 17-Aug-2011 | 7-Sep-2011 | 7-Oct-2011 | 26-Oct-2011 | 10-Nov-2011 |
| --- | --- | --- | --- | --- | --- | --- | --- | --- | --- | --- | --- | --- | --- | --- | --- | --- | --- | --- |
| OR_SIS | 3082/43 | 30.023 | 28.978 | 32.757 | 31.473 | 23.212 | 44.099 | 21.074 | - | 20.407 | 9.083 | - | - | - | - | 18.521 | 20.971 | - |
| OR_SIS | 3082/46 | 33.175 | 92.797 | 31.578 | 32.433 | 29.090 | 29.915 | 31.236 | 32.139 | 34.547 | 39.215 | 36.817 | 16.116 | 40.424 | 30.492 | - | - | - |
| OR_SIS | 3082/89 | 28.722 | 29.213 | 34.144 | 27.445 | 27.530 | 28.748 | 25.141 | 27.829 | 22.436 | 28.732 | 29.921 | 29.370 | 36.552 | 6.230 | 11.373 | 9.029 | 23.755 |
| OR_SIS | 3076/90 | 32.547 | 29.875 | 32.358 | 34.417 | 25.971 | 31.527 | 61.502 | 34.101 | 31.573 | 41.803 | 32.973 | 34.914 | 34.524 | 13.439 | 18.340 | 17.852 | 28.068 |
| OR_SIS | 3076/44 | 35.646 | 33.323 | 34.584 | 37.078 | 29.008 | 40.025 | 29.989 | 52.716 | 35.359 | 33.568 | 33.534 | 35.481 | 29.130 | 19.959 | 25.580 | 23.583 | 36.303 |
| WA_CAS | 1147/05 |  |  |  |  |  |  | 48.157 | 16.829 | 37.651 | 31.849 |  |  |  |  |  |  |  |
| WA_CAS | 1147/39 |  |  |  |  |  |  | 33.887 | 28.799 | 37.577 | 31.006 |  |  |  |  |  |  |  |
| WA_CAS | 1162/08 |  |  |  |  |  |  | 21.467 | 47.435 | 25.546 | 31.518 |  |  |  |  |  |  |  |
| WA_CAS | 1162/38 |  |  |  |  |  |  | 33.902 | 41.512 | 36.128 | - |  |  |  |  |  |  |  |
| WA_CST | 7202/10 |  |  |  |  |  |  | 32.091 | 22.661 | 39.999 | 34.478 |  |  |  |  |  |  |  |
| WA_CST | 7202/69 |  |  |  |  |  |  | 43.980 | 37.162 | 46.988 | 36.947 |  |  |  |  |  |  |  |
| WA_CST | 7205/12 |  |  |  |  |  |  | 32.994 | 26.437 | 25.881 | 30.879 |  |  |  |  |  |  |  |
| WA_CST | 7205/68 |  |  |  |  |  |  | 48.132 | 38.064 | 34.198 | 32.246 |  |  |  |  |  |  |  |
|  | *total* | *107 libraries* | | | | | | | | | | | | | | | | |

**Table S2:** Sampling intervals and Douglas-fir needles samples collected for the diurnal cycling study. Needles were collected at six hour intervals, starting at 2AM on September 8, 2011. For this experiment, transcriptome samples from independent needle RNA-Seq libraries were used to construct individual tree de novo references. Once the reference was constructed, reads were mapped back to determine counts per sampling interval for each tree. Upper table: the number of reads (in millions) obtained per sample are indicated. Lower table: SRA accession numbers for each sample and time point.

|  |  | Day 1 | | | | | | Day 2 | | | | | |
| --- | --- | --- | --- | --- | --- | --- | --- | --- | --- | --- | --- | --- | --- |
| Source | Tree | 2:00A | 6:00A | 10:00A | 14:00P | 18:00P | 22:00P | 2:00A | 6:00A | 10:00A | 14:00P | 18:00P | 22:00P |
| OR_SIS | 6870 | 30.612 | 24.035 | 17.460 | 26.832 | 30.912 | 32.821 | 27.378 | 34.579 | 32.471 | 35.853 | 30.427 | 29.894 |
| OR_SIS | 6871 | 18.386 | 28.973 | 10.460 | 27.983 | 24.261 | 30.846 | 30.757 | 30.391 | 27.878 | 35.410 | 36.540 | 46.269 |
| OR_SIS | 2096 | 29.086 | 31.347 | 27.009 | 13.319 | 18.825 | 47.227 | 40.042 | 26.401 | 32.634 | 32.375 | 41.709 | 42.864 |
| OR_SIS | 2098 | 31.965 | 31.482 | 31.099 | 29.756 | 27.387 | 37.827 | 24.765 | 26.145 | 25.086 | 53.355 | 30.119 | 27.482 |
| OR_SIS | 2486 | 33.175 | 36.258 | 44.597 | 21.573 | 72.435 | 20.144 | 26.269 | 77.155 | 33.528 | 33.785 | 28.570 | 43.339 |
| OR_SIS | 2491 | 31.518 | 44.706 | 37.529 | 34.705 | 24.496 | 27.571 | 68.616 | 18.566 | 42.746 | 38.335 | 35.269 | 24.445 |
|  | *total* | *72 libraries* | | | | | | | | | | | |

|  |  | Day 1 | | | | | | Day 2 | | | | | |
| --- | --- | --- | --- | --- | --- | --- | --- | --- | --- | --- | --- | --- | --- |
| Source | Tree | 2:00A | 6:00A | 10:00A | 14:00P | 18:00P | 22:00P | 2:00A | 6:00A | 10:00A | 14:00P | 18:00P | 22:00P |
| OR_SIS | 6870 | SRX1341340 | SRX1341341 | SRX1341342 | SRX1341343 | SRX1341344 | SRX1341345 | SRX1341346 | SRX1341347 | SRX1341348 | SRX1341349 | SRX1341350 | SRX1341351 |
| OR_SIS | 6871 | SRX1341352 | SRX1341353 | SRX1341319 | SRX1341354 | SRX1341355 | SRX1341356 | SRX1341357 | SRX1341358 | SRX1341359 | SRX1341360 | SRX1341361 | SRX1341362 |
| OR_SIS | 2096 | SRX1341321 | SRX1341363 | SRX1341323 | SRX1341325 | SRX1341364 | SRX1341365 | SRX1341366 | SRX1341367 | SRX1341368 | SRX1341369 | SRX1341370 | SRX1341371 |
| OR_SIS | 2098 | SRX1341372 | SRX1341327 | SRX1341329 | SRX1341331 | SRX1341373 | SRX1341374 | SRX1341375 | SRX1341376 | SRX1341377 | SRX1341378 | SRX1341379 | SRX1341380 |
| OR_SIS | 2486 | SRX1341381 | SRX1341382 | SRX1341383 | SRX1341384 | SRX1341385 | SRX1341386 | SRX1341387 | SRX1341332 | SRX1341388 | SRX1341389 | SRX1341390 | SRX1341391 |
| OR_SIS | 2491 | SRX1341392 | SRX1341393 | SRX1341394 | SRX1341395 | SRX1341396 | SRX1341397 | SRX1341335 | SRX1341337 | SRX1341339 | SRX1341398 | SRX1341399 | SRX1341400 |

**Table S3:** Individual trees and summary statistics for all libraries used to construct individual tree de novo transcriptome references. For this experiment, transcriptome samples from between 4 and 16 independent needle RNA-Seq libraries were used to construct de novo references.

| Source | Family/ID | Experiment | Input libraries | Untrimmed reads | Untrimmed bases | Trimmed reads | Trimmed bases | Mean trimmed length (bp) |
| --- | --- | --- | --- | --- | --- | --- | --- | --- |
| OR_SIS | /6870 | Diurnal | 12 | 353,274,260 | 35,680,700,260 | 338,115,080 | 29,336,995,483 | 86.8 |
| OR_SIS | /6871 | Diurnal | 12 | 348,155,561 | 35,163,711,661 | 333,757,534 | 29,202,834,770 | 87.5 |
| OR_SIS | /2096 | Diurnal | 12 | 430,079,575 | 43,438,037,075 | 407,483,919 | 34,810,655,854 | 85.4 |
| OR_SIS | /2098 | Diurnal | 12 | 456,198,139 | 46,076,012,039 | 444,170,407 | 37,876,271,982 | 85.3 |
| OR_SIS | /2486 | Diurnal | 12 | 485,482,381 | 49,033,720,481 | 466,929,572 | 40,794,581,780 | 87.4 |
| OR_SIS | /2491 | Diurnal | 12 | 504,477,902 | 50,952,268,102 | 484,526,954 | 42,130,526,581 | 87.0 |
| OR_SIS | 3082/43 | Annual | 11 | 280,596,851 | 28,340,281,951 | 272,545,294 | 24,739,094,086 | 90.8 |
| OR_SIS | 3082/46 | Annual | 14 | 588,635,169 | 59,452,152,069 | 555,638,025 | 44,731,980,025 | 80.5 |
| OR_SIS | 3082/89 | Annual | 17 | 435,715,465 | 44,007,261,965 | 416,756,566 | 35,742,306,842 | 85.8 |
| OR_SIS | 3076/44 | Annual | 17 | 598,221,484 | 60,420,369,884 | 573,792,151 | 49,673,552,256 | 86.6 |
| OR_SIS | 3076/90 | Annual | 17 | 547,124,445 | 55,259,568,945 | 520,118,937 | 44,590,537,450 | 85.7 |
| WA_CAS | 1147/05 | Spring | 4 | 134,486,117 | 13,583,097,817 | 126,126,731 | 10,675,283,471 | 84.6 |
| WA_CAS | 1147/39 | Spring | 3 | 131,269,557 | 13,258,225,257 | 122,090,800 | 10,078,425,387 | 82.5 |
| WA_CAS | 1162/08 | Spring | 4 | 125,965,686 | 12,722,534,286 | 118,148,550 | 9,881,829,674 | 83.6 |
| WA_CAS | 1162/38 | Spring | 4 | 100,350,323 | 10,135,382,623 | 94,023,054 | 7,919,208,706 | 84.2 |
| WA_CST | 7202/10 | Spring | 4 | 129,229,211 | 13,052,150,311 | 120,862,154 | 10,214,613,893 | 84.5 |
| WA_CST | 7202/69 | Spring | 4 | 165,076,806 | 16,672,757,406 | 152,667,375 | 12,539,794,728 | 82.1 |
| WA_CST | 7205/12 | Spring | 4 | 116,190,288 | 11,735,219,088 | 109,052,038 | 9,214,201,849 | 84.5 |
| WA_CST | 7205/68 | Spring | 4 | 152,640,485 | 15,416,688,985 | 141,612,028 | 11,678,039,560 | 82.5 |
| *Sum* |  |  |  | *6,083,169,705* | *614,400,140,205* | *5,798,417,169* | *495,830,734,377* |  |

**Table S4:** Individual tree summary statistics for Trinity de novo transcriptome references. For this experiment, transcriptome samples from between 4 and 16 independent needle RNA-Seq libraries were used to construct de novo references. Summary metrics (cumulative sequence; N50; longest transcript) were calculated using the longest Trinity Component for transcripts meeting a minimum length criterion, either 200 bp (for Trinity assemblies) or 300 bp (for clustered and reduced references).

| Source | Family/ID | Experiment | Input bases | Trinity Components | Trinity Sub-components | Trinity Sequences | Cumulative Sequence (bp) | N_50_ | Longest Transcript | Shortest Transcript |
| --- | --- | --- | --- | --- | --- | --- | --- | --- | --- | --- |
| OR_SIS | /6870 | Diurnal | 29,336,995,483 | 111,583 | 112,312 | 159,669 | 138,784,089 | 1,024 | 14,079 | 200 |
| OR_SIS | /6871 | Diurnal | 29,202,834,770 | 109,268 | 110,104 | 162,810 | 146,952,399 | 1,059 | 14,166 | 200 |
| OR_SIS | /2096 | Diurnal | 34,810,655,854 | 98,883 | 99,726 | 157,040 | 149,114,688 | 1,284 | 22,686 | 200 |
| OR_SIS | /2098 | Diurnal | 37,876,271,982 | 125,293 | 126,312 | 183,818 | 166,479,295 | 1,057 | 35,465 | 200 |
| OR_SIS | /2486 | Diurnal | 40,794,581,780 | 108,198 | 109,127 | 161,778 | 153,457,099 | 1,140 | 37,431 | 200 |
| OR_SIS | /2491 | Diurnal | 42,130,526,581 | 101,174 | 102,132 | 153,891 | 144,711,451 | 1,273 | 22,689 | 200 |
| OR_SIS | 3082/43 | Annual | 24,739,094,086 | 93,351 | 94,233 | 144,622 | 142,969,456 | 1,373 | 26,701 | 200 |
| OR_SIS | 3082/46 | Annual | 44,731,980,025 | 125,610 | 126,714 | 192,468 | 172,119,131 | 1,026 | 18,054 | 200 |
| OR_SIS | 3082/89 | Annual | 35,742,306,842 | 111,595 | 112,578 | 170,920 | 158,510,984 | 1,136 | 21,152 | 200 |
| OR_SIS | 3076/44 | Annual | 49,673,552,256 | 126,355 | 127,533 | 195,054 | 177,527,065 | 1,051 | 22,668 | 200 |
| OR_SIS | 3076/90 | Annual | 44,590,537,450 | 115,601 | 116,633 | 178,101 | 164,957,053 | 1,139 | 16,412 | 200 |
| WA_CAS | 1147/05 | Spring | 10,675,283,471 | 55,877 | 56,372 | 85,851 | 86,513,576 | 1,512 | 18,445 | 200 |
| WA_CAS | 1147/39 | Spring | 10,078,425,387 | 53,135 | 53,561 | 82,337 | 83,444,189 | 1,518 | 20,329 | 200 |
| WA_CAS | 1162/08 | Spring | 9,881,829,674 | 55,858 | 56,277 | 86,718 | 86,594,754 | 1,512 | 24,082 | 200 |
| WA_CAS | 1162/38 | Spring | 7,919,208,706 | 47,976 | 48,277 | 70,749 | 69,149,722 | 1,525 | 20,356 | 200 |
| WA_CST | 7202/10 | Spring | 10,214,613,893 | 52,230 | 52,607 | 77,833 | 77,784,631 | 1,547 | 19,713 | 200 |
| WA_CST | 7202/69 | Spring | 12,539,794,728 | 54,857 | 55,368 | 86,694 | 88,670,092 | 1,554 | 17,657 | 200 |
| WA_CST | 7205/12 | Spring | 9,214,201,849 | 49,605 | 49,986 | 74,970 | 75,246,197 | 1,553 | 19,491 | 200 |
| WA_CST | 7205/68 | Spring | 11,678,039,560 | 60,097 | 60,584 | 95,331 | 99,111,242 | 1,521 | 20,934 | 200 |
| *Means* |  |  | *26,096,354,440* | *87,187* | *87,918* | *132,666* | *125,373,532* | *1,305* | *21,711* | *200* |
| **Total clustered pan-transcriptome reference** | | | | | | **199,623** | **176,487,371** | **1,316** | **37,431** | **300** |
| **Pan-transcriptome “plant-only” transcripts** | | | | | | **173,882** | **165,181,440** | **1,410** | **37,431** | **300** |

**Table S5:** Summaries for diurnal transcripts shown in Figure 3. Each transcript is identified by the Douglas-fir pan-transcriptome accession number, the best TBLASTX match from Arabidopsis thaliana (from Mercator database; http://mapman.gabipd.org/web/guest/app/mercator), the A. thaliana gene name and Mercator Bin name, Mercator annotation description, the phase in clock hours, and the P value from JTK-cycle. Genes are ordered in increasing order of phase, starting at midnight (0:00 AM). Similar annotations are provided for all transcripts on the Dendrome website (https://dendrome.ucdavis.edu/treegenes/species/species_detail.php?id=102/) under the link “Pseudotsuga menziesii Transcriptome”

| **Douglas-fir transcript** | ***Arabidopsis* match (Mercator)** | **Gene Name (Mercator Bin)** | **Mercator Description** | **Phase (clock hour)** | **BH.Q**  **(JTK-cycle)** |
| --- | --- | --- | --- | --- | --- |
| T010614 | AT1G62200.1 | PTR2  (transport.amino acids) | (at2g02040 : 793.0) Encodes a di- and tri-peptide transporter that recognizes a variety of different amino acid combinations. Expression of the transcripts for this gene can be detected in the embryo through in situ hybridization. This protein does not have nitrate transporter activity based on oocyte transport assays.; peptide transporter 2 (PTR2); FUNCTIONS IN: dipeptide transporter activity, high affinity oligopeptide transporter activity, tripeptide transporter activity, peptide transporter activity, transporter activity; INVOLVED IN: dipeptide transport, tripeptide transport, peptide transport; LOCATED IN: vacuolar membrane, plasma membrane, vacuole, plant-type vacuole; EXPRESSED IN: 24 plant structures; EXPRESSED DURING: 13 growth stages; CONTAINS InterPro DOMAIN/s: PTR2 family proton/oligopeptide symporter, conserved site (InterPro:IPR018456), Oligopeptide transporter (InterPro:IPR000109), Major facilitator superfamily, general substrate transporter (InterPro:IPR016196); BEST Arabidopsis thaliana protein match is: Major facilitator superfamily protein (TAIR:AT1G62200.1); Has 8080 Blast hits to 7668 proteins in 1494 species: Archae - 0; Bacteria - 3960; Metazoa - 799; Fungi - 488; Plants - 2224; Viruses - 0; Other Eukaryotes - 609 (source: NCBI BLink). & (reliability: 1586.0) & (original description: no original description) | 00:00 | 1.29e-16 |
| T005329 | AT5G37260.1 | RVE1  (RNA.regulation of transcription.MYB-related transcription factor family) | (at5g17300 : 121.0) Myb-like transcription factor that regulates hypocotyl growth by regulating free auxin levels in a time-of-day specific manner.; REVEILLE 1 (RVE1); CONTAINS InterPro DOMAIN/s: SANT, DNA-binding (InterPro:IPR001005), Homeodomain-like (InterPro:IPR009057), Myb, DNA-binding (InterPro:IPR014778), HTH transcriptional regulator, Myb-type, DNA-binding (InterPro:IPR017930), Myb-like DNA-binding domain, SHAQKYF class (InterPro:IPR006447); BEST Arabidopsis thaliana protein match is: Homeodomain-like superfamily protein (TAIR:AT5G37260.1); Has 1557 Blast hits to 1521 proteins in 166 species: Archae - 0; Bacteria - 3; Metazoa - 201; Fungi - 29; Plants - 1060; Viruses - 9; Other Eukaryotes - 255 (source: NCBI BLink). & (reliability: 242.0) & (original description: no original description) | 00:00 | 2.82e-18 |
| t046935 | AT4G02890.2 | UBQ11 (protein.degradation. ubiquitin.ubiquitin) | (at4g05050 : 444.0) polyubiquitin gene, belongs to a subtype group with UBQ10 and UBQ14. Various ecotypes of Arabidopsis have different numbers of ubiquitin repeats within this gene.; ubiquitin 11 (UBQ11); INVOLVED IN: protein modification process, response to salt stress, ubiquitin-dependent protein catabolic process; LOCATED IN: intracellular; EXPRESSED IN: 24 plant structures; EXPRESSED DURING: 15 growth stages; CONTAINS InterPro DOMAIN/s: Ubiquitin subgroup (InterPro:IPR019956), Ubiquitin conserved site (InterPro:IPR019954), Ubiquitin (InterPro:IPR000626), Ubiquitin supergroup (InterPro:IPR019955); BEST Arabidopsis thaliana protein match is: Ubiquitin family protein (TAIR:AT4G02890.2). & (p69326\|ubiq_wheat : 150.0) Ubiquitin - Triticum aestivum (Wheat) & (reliability: 888.0) & (original description: no original description) | 02:00 | 2.55e-15 |
| t005030 | AT5G62430.1 | CDF3  (RNA.regulation of transcription.C2C2(Zn) DOF zinc finger family) | (at3g47500 : 192.0) Dof-type zinc finger domain-containing protein, identical to H-protein promoter binding factor-2a GI:3386546 from (Arabidopsis thaliana). Interacts with LKP2 and FKF1, but its overexpression does not change flowering time under short or long day conditions.; cycling DOF factor 3 (CDF3); CONTAINS InterPro DOMAIN/s: Zinc finger, Dof-type (InterPro:IPR003851); BEST Arabidopsis thaliana protein match is: cycling DOF factor 1 (TAIR:AT5G62430.1); Has 1160 Blast hits to 1145 proteins in 79 species: Archae - 0; Bacteria - 0; Metazoa - 13; Fungi - 10; Plants - 1084; Viruses - 0; Other Eukaryotes - 53 (source: NCBI BLink). & (o24463\|pbf_maize : 98.6) Dof zinc finger protein PBF (Prolamin box-binding factor) - Zea mays (Maize) & (reliability: 384.0) & (original description: no original description) | 04:00 | 3.55e-23 |
| t005024 | AT5G57380.1 | VEL1  (development. Unspecified) | (at4g30200 : 383.0) Encodes a protein with similarity to VRN5 and VIN3.Contains both a fibronectin III and PHD finger domain. VEL1 is a part of a polycomb repressive complex (PRC2) that is involved in epigenetic silencing of the FLC flowering locus.; vernalization5/VIN3-like (VEL1); FUNCTIONS IN: molecular_function unknown; INVOLVED IN: vegetative to reproductive phase transition of meristem; LOCATED IN: PcG protein complex; EXPRESSED IN: 24 plant structures; EXPRESSED DURING: 15 growth stages; CONTAINS InterPro DOMAIN/s: Protein of unknown function DUF1423, plant (InterPro:IPR004082), Fibronectin, type III-like fold (InterPro:IPR008957), Fibronectin, type III (InterPro:IPR003961); BEST Arabidopsis thaliana protein match is: Fibronectin type III domain-containing protein (TAIR:AT5G57380.1). & (reliability: 736.0) & (original description: no original description) | 04:00 | 2.95e-11 |
| t001299 | AT1G01060.3 | CCA1  (RNA.regulation of transcription.MYB-related transcription factor family) | (at2g46830 : 140.0) Encodes a transcriptional repressor that performs overlapping functions with LHY in a regulatory feedback loop that is closely associated with the circadian oscillator of Arabidopsis. Binds to the evening element in the promoter of TOC1 and represses TOC1 transcription. CCA1 and LHY colocalize in the nucleus and form heterodimers in vivo. CCA1 and LHY function synergistically in regulating circadian rhythms of Arabidopsis.; circadian clock associated 1 (CCA1); BEST Arabidopsis thaliana protein match is: Homeodomain-like superfamily protein (TAIR:AT1G01060.3); Has 2694 Blast hits to 869 proteins in 218 species: Archae - 5; Bacteria - 81; Metazoa - 344; Fungi - 210; Plants - 198; Viruses - 7; Other Eukaryotes - 1849 (source: NCBI BLink). & (reliability: 280.0) & (original description: no original description) | 04:00 | 2.47e-17 |
| T007671 | AT4G11110.1 | COP1  (development.  Unspecified) | (at2g32950 : 836.0) Represses photomorphogenesis and induces skotomorphogenesis in the dark. Contains a ring finger zinc-binding motif, a coiled-coil domain, and several WD-40 repeats, similar to G-beta proteins. The C-terminus has homology to TAFII80, a subunit of the TFIID component of the RNA polymerase II of Drosophila. Nuclear localization in the dark and cytoplasmic in the light.; CONSTITUTIVE PHOTOMORPHOGENIC 1 (COP1); CONTAINS InterPro DOMAIN/s: WD40 repeat 2 (InterPro:IPR019782), Zinc finger, RING-type, conserved site (InterPro:IPR017907), Zinc finger, RING-type (InterPro:IPR001841), WD40 repeat, conserved site (InterPro:IPR019775), WD40 repeat (InterPro:IPR001680), WD40 repeat-like-containing domain (InterPro:IPR011046), WD40-repeat-containing domain (InterPro:IPR017986), Zinc finger, C3HC4 RING-type (InterPro:IPR018957), WD40/YVTN repeat-like-containing domain (InterPro:IPR015943), WD40 repeat, subgroup (InterPro:IPR019781); BEST Arabidopsis thaliana protein match is: SPA1-related 2 (TAIR:AT4G11110.1); Has 42218 Blast hits to 27649 proteins in 756 species: Archae - 30; Bacteria - 4454; Metazoa - 17716; Fungi - 8958; Plants - 5306; Viruses - 46; Other Eukaryotes - 5708 (source: NCBI BLink). & (p93471\|cop1_pea : 819.0) E3 ubiquitin ligase protein COP1 (EC 6.3.2.-) (Constitutive photomorphogenesis protein 1) - Pisum sativum (Garden pea) & (reliability: 1672.0) & (original description: no original description) | 07:00 | 1.34e-13 |
| t020254 |  | not assigned.unknown | no hits & (original description: no original description) | 10:00 | 1.32e-23 |
| T021120 | AT4G20370.1 | FT  (development. Unspecified) | (at1g65480 : 195.0) FT, together with LFY, promotes flowering and is antagonistic with its homologous gene, TERMINAL FLOWER1 (TFL1). FT is expressed in leaves and is induced by long day treatment. Either the FT mRNA or protein is translocated to the shoot apex where it induces its own expression. Recent data suggests that FT protein acts as a long-range signal. FT is a target of CO and acts upstream of SOC1.; FLOWERING LOCUS T (FT); FUNCTIONS IN: phosphatidylethanolamine binding, protein binding; INVOLVED IN: photoperiodism, flowering, positive regulation of flower development, regulation of flower development; LOCATED IN: nucleus, cytoplasm; EXPRESSED IN: 14 plant structures; EXPRESSED DURING: 7 growth stages; CONTAINS InterPro DOMAIN/s: Phosphatidylethanolamine-binding, conserved site (InterPro:IPR001858), Phosphatidylethanolamine-binding protein PEBP (InterPro:IPR008914); BEST Arabidopsis thaliana protein match is: PEBP (phosphatidylethanolamine-binding protein) family protein (TAIR:AT4G20370.1); Has 2182 Blast hits to 2182 proteins in 306 species: Archae - 0; Bacteria - 0; Metazoa - 594; Fungi - 140; Plants - 1404; Viruses - 3; Other Eukaryotes - 41 (source: NCBI BLink). & (q9xh44\|cet1_tobac : 193.0) CEN-like protein 1 - Nicotiana tabacum (Common tobacco) & (reliability: 390.0) & (original description: no original description) | 12:00 | 2.21e-9 |
| T027724 | AT1G72630 | ELF4-like  (not assigned. unknown) | (at1g72630 : 139.0) ELF4-like 2 (ELF4-L2); CONTAINS InterPro DOMAIN/s: Protein of unknown function DUF1313 (InterPro:IPR009741); BEST Arabidopsis thaliana protein match is: ELF4-like 4 (TAIR:AT1G17455.2); Has 149 Blast hits to 148 proteins in 34 species: Archae - 0; Bacteria - 0; Metazoa - 0; Fungi - 0; Plants - 148; Viruses - 0; Other Eukaryotes - 1 (source: NCBI BLink). & (reliability: 278.0) & (original description: no original description) | 12:00 | 1.90e-6 |
| T001582 | AT1G22770 | GI  (development. unspecified) | (q9awl7\|gigan_orysa : 1074.0) Protein GIGANTEA - Oryza sativa (Rice) & (at1g22770 : 1031.0) Together with CONSTANTS (CO) and FLOWERING LOCUS T (FT), GIGANTEA promotes flowering under long days in a circadian clock-controlled flowering pathway. GI acts earlier than CO and FT in the pathway by increasing CO and FT mRNA abundance. Located in the nucleus. Regulates several developmental processes, including photoperiod-mediated flowering, phytochrome B signaling, circadian clock, carbohydrate metabolism, and cold stress response. The genes transcription is controlled by the circadian clock and it is post-transcriptionally regulated by light and dark. Forms a complex with FKF1 on the CO promoter to regulate CO expression.; GIGANTEA (GI); Has 351 Blast hits to 349 proteins in 54 species: Archae - 0; Bacteria - 0; Metazoa - 0; Fungi - 0; Plants - 351; Viruses - 0; Other Eukaryotes - 0 (source: NCBI BLink). & (reliability: 2062.0) & (original description: no original description) | 18:00 | 3.90e-9 |
| T004201 | AT2G46830.1 | LHY  (RNA.regulation of transcription.MYB-related transcription factor family) | (at1g01060 : 116.0) LHY encodes a myb-related putative transcription factor involved in circadian rhythm along with another myb transcription factor CCA1; LATE ELONGATED HYPOCOTYL (LHY); FUNCTIONS IN: DNA binding, sequence-specific DNA binding transcription factor activity; INVOLVED IN: in 11 processes; EXPRESSED IN: 22 plant structures; EXPRESSED DURING: 13 growth stages; BEST Arabidopsis thaliana protein match is: circadian clock associated 1 (TAIR:AT2G46830.1). & (reliability: 232.0) & (original description: no original description) | 18:00 | 3.88e-10 |
| T005033 | AT2G18915.2 | ZTL  (protein.degradation. ubiquitin.E3.SCF.FBOX) | (at5g57360 : 907.0) Encodes clock-associated PAS protein ZTL; Also known as FKF1-like protein 2 or ADAGIO1(ADO1). A protein containing a PAS domain ZTL contributes to the plant fitness (carbon fixation, biomass) by influencing the circadian clock period. ZTL is the F-box component of an SCF complex implicated in the degradation of TOC1.; ZEITLUPE (ZTL); CONTAINS InterPro DOMAIN/s: F-box domain, cyclin-like (InterPro:IPR001810), Kelch repeat type 1 (InterPro:IPR006652), Galactose oxidase/kelch, beta-propeller (InterPro:IPR011043), PAS fold (InterPro:IPR013767), Kelch repeat type 2 (InterPro:IPR011498), PAS (InterPro:IPR000014), Kelch-type beta propeller (InterPro:IPR015915); BEST Arabidopsis thaliana protein match is: LOV KELCH protein 2 (TAIR:AT2G18915.2); Has 8358 Blast hits to 5610 proteins in 807 species: Archae - 89; Bacteria - 2025; Metazoa - 1889; Fungi - 901; Plants - 2129; Viruses - 0; Other Eukaryotes - 1325 (source: NCBI BLink). & (q5z8k3\|ado1_orysa : 893.0) Adagio-like protein 1 - Oryza sativa (Rice) & (reliability: 1814.0) & (original description: no original description) | 20:00 | 1.37e-7 |
| T012828 | AT5G59570.1 | LUX  (RNA.regulation of transcription.G2-like transcription factor family, GARP) | (at3g46640 : 221.0) Encodes a myb family transcription factor with a single Myb DNA-binding domain (type SHAQKYF) that is unique to plants and is essential for circadian rhythms, specifically for transcriptional regulation within the circadian clock. LUX is required for normal rhythmic expression of multiple clock outputs in both constant light and darkness. It is coregulated with TOC1 and seems to be repressed by CCA1 and LHY by direct binding of these proteins to the evening element in the LUX promoter.; PHYTOCLOCK 1 (PCL1); CONTAINS InterPro DOMAIN/s: Myb, DNA-binding (InterPro:IPR014778), Homeodomain-like (InterPro:IPR009057), Homeodomain-related (InterPro:IPR012287), Myb-like DNA-binding domain, SHAQKYF class (InterPro:IPR006447), HTH transcriptional regulator, Myb-type, DNA-binding (InterPro:IPR017930); BEST Arabidopsis thaliana protein match is: Homeodomain-like superfamily protein (TAIR:AT5G59570.1). & (reliability: 442.0) & (original description: no original description) | 20:00 | 1.68e-6 |
| T039766 | AT5G60100.3 | TOC1  (RNA.regulation of transcription.Psudo ARR transcription factor family) | (at5g61380 : 201.0) Pseudo response regulator involved in the generation of circadian rhythms. TOC1 appears to shorten the period of circumnutation speed. TOC1 contributes to the plant fitness (carbon fixation, biomass) by influencing the circadian clock period. PRR3 may increase the stability of TOC1 by preventing interactions between TOC1 and the F-box protein ZTL. Expression of TOC1 is correlated with rhythmic changes in chromatin organization.; TIMING OF CAB EXPRESSION 1 (TOC1); CONTAINS InterPro DOMAIN/s: CheY-like (InterPro:IPR011006), Signal transduction response regulator, receiver domain (InterPro:IPR001789), CCT domain (InterPro:IPR010402); BEST Arabidopsis thaliana protein match is: pseudo-response regulator 3 (TAIR:AT5G60100.3); Has 1807 Blast hits to 1807 proteins in 277 species: Archae - 0; Bacteria - 0; Metazoa - 736; Fungi - 347; Plants - 385; Viruses - 0; Other Eukaryotes - 339 (source: NCBI BLink). & (q689g9\|prr1_orysa : 188.0) Two-component response regulator-like PRR1 (Pseudo-response regulator 1) (OsPRR1) - Oryza sativa (Rice) & (reliability: 402.0) & (original description: no original description) | 20:00 | 5.26e-11 |

**Table S6:** Summaries for annual transcripts shown in Figure 5. Each transcript is identified by the Douglas-fir pan-transcriptome accession number, the best TBLASTX match from Arabidopsis thaliana (from Mercator database; http://mapman.gabipd.org/web/guest/app/mercator), the A. thaliana gene name and Mercator Bin name, Mercator annotation description, the phase in experiment days and month, and the P value from JTK-cycle. Genes are ordered in increasing order of phase, starting at November. Similar annotations are provided for all transcripts on the Dendrome website (https://dendrome.ucdavis.edu/treegenes/species/species_detail.php?id=102/) under the link “Pseudotsuga menziesii Transcriptome”

| **Douglas-fir transcript** | ***Arabidopsis* match (Mercator)** | **Gene Name (Mercator bin name)** | **Mercator Description** | **Phase_365_, Julian Day (month)** | **BH.Q**  **(JTK-cycle)** |
| --- | --- | --- | --- | --- | --- |
| psme_t007714 | AT1G12860.1 | ice1 (RNA.regulation of transcription.bHLH,Basic Helix-Loop-Helix family) | (at3g26744 : 322.0) Encodes a MYC-like bHLH transcriptional activator that binds specifically to the MYC recognition sequences in the CBF3 promoter. Mutants are defective in cold-regulated gene expression. Cold stress triggers protein degradation of nuclear GFPICE1 protein, and the RING finger protein HOS1 is required. Sumoylation of ICE1 controls CBF3/DREB1A expression and freezing tolerance.; INDUCER OF CBF EXPRESSION 1 (ICE1); CONTAINS InterPro DOMAIN/s: Helix-loop-helix DNA-binding domain (InterPro:IPR001092), Helix-loop-helix DNA-binding (InterPro:IPR011598); BEST Arabidopsis thaliana protein match is: basic helix-loop-helix (bHLH) DNA-binding superfamily protein (TAIR:AT1G12860.1); Has 2623 Blast hits to 2616 proteins in 144 species: Archae - 0; Bacteria - 0; Metazoa - 19; Fungi - 30; Plants - 2572; Viruses - 0; Other Eukaryotes - 2 (source: NCBI BLink). & (p13027\|arrs_maize : 62.8) Anthocyanin regulatory R-S protein - Zea mays (Maize) & (reliability: 644.0) & (original description: no original description) | 321.8  (Nov) | 3.11e-10 |
| psme_t001547 | AT4G10760.1 | RNA.regulation of transcription.putative transcription regulator | (at4g09980 : 550.0) EMBRYO DEFECTIVE 1691 (EMB1691); FUNCTIONS IN: S-adenosylmethionine-dependent methyltransferase activity, methyltransferase activity, nucleic acid binding; INVOLVED IN: embryo development ending in seed dormancy; EXPRESSED IN: 24 plant structures; EXPRESSED DURING: 13 growth stages; CONTAINS InterPro DOMAIN/s: DNA methylase, N-6 adenine-specific, conserved site (InterPro:IPR002052), MT-A70 (InterPro:IPR007757); BEST Arabidopsis thaliana protein match is: mRNAadenosine methylase (TAIR:AT4G10760.1); Has 29929 Blast hits to 18009 proteins in 1024 species: Archae - 39; Bacteria - 4517; Metazoa - 16101; Fungi - 2453; Plants - 1900; Viruses - 259; Other Eukaryotes - 4660 (source: NCBI BLink). & (q2hvd6\|mta70_medtr : 119.0) Putative N6-adenosine-methyltransferase MT-A70-like (EC 2.1.1.62) - Medicago truncatula (Barrel medic) & (reliability: 1100.0) & (original description: no original description) | 343.4  (Dec) | 5.68e-7 |
| psme_t070199 | AT4G14690.1 | early light-inducible proteins, *ELIP*  (signalling.light) | (at3g22840 : 162.0) Encodes an early light-inducible protein.; EARLY LIGHT-INDUCABLE PROTEIN (ELIP1); BEST Arabidopsis thaliana protein match is: Chlorophyll A-B binding family protein (TAIR:AT4G14690.1); Has 319 Blast hits to 319 proteins in 50 species: Archae - 0; Bacteria - 5; Metazoa - 0; Fungi - 0; Plants - 259; Viruses - 0; Other Eukaryotes - 55 (source: NCBI BLink). & (p11432\|eli_pea : 153.0) Early light-induced protein, chloroplast precursor (ELIP) - Pisum sativum (Garden pea) & (reliability: 324.0) & (original description: no original description) | 13.8  (Jan) | 5.65e-12 |
| psme_t005652 | unknown | Unknown (not assigned.unknown) | no hits & (original description: no original description) | 32.3  (Feb) | 1.02e-10 |
| psme_t024224 | AT3G01210.1 | RNA-binding (RRM/RBD/RNP motifs) family protein | (at1g14340 : 247.0) RNA-binding (RRM/RBD/RNP motifs) family protein; FUNCTIONS IN: oxidoreductase activity, nucleotide binding, nucleic acid binding; INVOLVED IN: oxidation reduction; EXPRESSED IN: 22 plant structures; EXPRESSED DURING: 13 growth stages; CONTAINS InterPro DOMAIN/s: Aldo/keto reductase (InterPro:IPR001395), RNA recognition motif, RNP-1 (InterPro:IPR000504), Nucleotide-binding, alpha-beta plait (InterPro:IPR012677); BEST Arabidopsis thaliana protein match is: RNA-binding (RRM/RBD/RNP motifs) family protein (TAIR:AT3G01210.1); Has 332 Blast hits to 332 proteins in 76 species: Archae - 0; Bacteria - 0; Metazoa - 0; Fungi - 92; Plants - 229; Viruses - 0; Other Eukaryotes - 11 (source: NCBI BLink). & (reliability: 494.0) & (original description: no original description) | 70.9  (Mar) | 3.22e-9 |
| psme_t006273 | at2G21710 | Mitochondrial transcription termination factor family (RNA.regulation of transcription.unclassified) | (at1g78930 : 150.0) Mitochondrial transcription termination factor family protein; FUNCTIONS IN: molecular_function unknown; INVOLVED IN: biological_process unknown; LOCATED IN: chloroplast; EXPRESSED IN: 22 plant structures; EXPRESSED DURING: 13 growth stages; CONTAINS InterPro DOMAIN/s: Mitochodrial transcription termination factor-related (InterPro:IPR003690); BEST Arabidopsis thaliana protein match is: Mitochondrial transcription termination factor family protein (TAIR:AT2G21710.1); Has 1485 Blast hits to 916 proteins in 85 species: Archae - 0; Bacteria - 0; Metazoa - 111; Fungi - 0; Plants - 1282; Viruses - 0; Other Eukaryotes - 92 (source: NCBI BLink). & (reliability: 300.0) & (original description: no original description) | 93.7  (Apr) | 3.86e-9 |
| psme_t010809 | AT5G03330.2 | (protein.degradation.cysteine protease) | (at5g04250 : 305.0) Cysteine proteinases superfamily protein; FUNCTIONS IN: cysteine-type peptidase activity; INVOLVED IN: biological_process unknown; LOCATED IN: cellular_component unknown; EXPRESSED IN: 24 plant structures; EXPRESSED DURING: 15 growth stages; CONTAINS InterPro DOMAIN/s: Ovarian tumour, otubain (InterPro:IPR003323); BEST Arabidopsis thaliana protein match is: Cysteine proteinases superfamily protein (TAIR:AT5G03330.2); Has 30201 Blast hits to 17322 proteins in 780 species: Archae - 12; Bacteria - 1396; Metazoa - 17338; Fungi - 3422; Plants - 5037; Viruses - 0; Other Eukaryotes - 2996 (source: NCBI BLink). & (reliability: 610.0) & (original description: no original description) | 127.9  (May) | 3.53e-7 |
| psme_t023177 | AT5G45280.2 | (cell wall. Pectin esterases .acetyl esterase) | (at4g19410 : 127.0) Pectinacetylesterase family protein; FUNCTIONS IN: actin binding, carboxylesterase activity; INVOLVED IN: cytoskeleton organization; LOCATED IN: plant-type cell wall; EXPRESSED IN: 23 plant structures; EXPRESSED DURING: 13 growth stages; CONTAINS InterPro DOMAIN/s: Pectinacetylesterase (InterPro:IPR004963), Profilin/allergen (InterPro:IPR002097); BEST Arabidopsis thaliana protein match is: Pectinacetylesterase family protein (TAIR:AT5G45280.2).: 254.0) & (original description: no original description) | 174.6  (Jun) | 2.42e-10 |
| psme_t044566 | AT3G58680.1 | hormone metabolism.ethylene.induced-regulated-responsive-activated | (at3g24500 : 197.0) One of three genes in A. thaliana encoding multiprotein bridging factor 1, a highly conserved transcriptional coactivator. May serve as a bridging factor between a bZIP factor and TBP. Its expression is specifically elevated in response to pathogen infection, salinity, drought, heat, hydrogen peroxide, and application of abscisic acid or salicylic acid. Constitutive expression enhances the tolerance of transgenic plants to various biotic and abiotic stresses.; multiprotein bridging factor 1C (MBF1C); FUNCTIONS IN: transcription coactivator activity, DNA binding, sequence-specific DNA binding transcription factor activity; INVOLVED IN: in 7 processes; LOCATED IN: nucleolus; EXPRESSED IN: 24 plant structures; EXPRESSED DURING: 14 growth stages; CONTAINS InterPro DOMAIN/s: Multiprotein bridging factor 1, N-terminal (InterPro:IPR013729); BEST Arabidopsis thaliana protein match is: multiprotein bridging factor 1B (TAIR:AT3G58680.1). & (reliability: 394.0) & (original description: no original description) | 196.3  (Jul) | 6.57e-6 |
| psme_t012941 | AT5G47970.2 | FMN-linked oxidoreductases superfamily protein (N-metabolism.misc) | (at3g63510 : 457.0) FMN-linked oxidoreductases superfamily protein; FUNCTIONS IN: tRNA dihydrouridine synthase activity, FAD binding, catalytic activity; INVOLVED IN: regulation of nitrogen utilization, tRNA processing, oxidation reduction, metabolic process; EXPRESSED IN: 18 plant structures; EXPRESSED DURING: 13 growth stages; CONTAINS InterPro DOMAIN/s: Aldolase-type TIM barrel (InterPro:IPR013785), tRNA-dihydrouridine synthase (InterPro:IPR001269); BEST Arabidopsis thaliana protein match is: Aldolase-type TIM barrel family protein (TAIR:AT5G47970.2); Has 11664 Blast hits to 11660 proteins in 2553 species: Archae - 58; Bacteria - 8007; Metazoa - 234; Fungi - 152; Plants - 146; Viruses - 0; Other Eukaryotes - 3067 (source: NCBI BLink). & (reliability: 848.0) & (original description: no original description) | 213.8  (Aug) | 2.21e-11 |
| psme_t023214 | AT1G14980.1 | (protein.folding) | (at5g20720 : 303.0) Encodes a chloroplast co-chaperonin with similarity to CPN21 from spinach, E.coli GroES.; chaperonin 20 (CPN20); CONTAINS InterPro DOMAIN/s: Chaperonin Cpn10 (InterPro:IPR020818), GroES-like (InterPro:IPR011032), Chaperonin Cpn10, conserved site (InterPro:IPR018369), Chaperonin 21, chloroplast (InterPro:IPR017416), Chaperonin Cpn10, subgroup (InterPro:IPR001476); BEST Arabidopsis thaliana protein match is: chaperonin 10 (TAIR:AT1G14980.1). & (q02073\|ch10c_spiol : 285.0) 20 kDa chaperonin, chloroplast precursor (Protein Cpn21) (Chloroplast protein Cpn10) (Chloroplast chaperonin 10) (Ch-CPN10) - Spinacia oleracea (Spinach) & (reliability: 606.0) & (original description: no original description) | 244.6  (Sep) | 5.67e-8 |
| psme_t011948 | AT4G29190.1 | RNA.regulation of transcription.C3H zinc finger family | (at2g19810 : 249.0) CCCH-type zinc finger family protein; CONTAINS InterPro DOMAIN/s: Zinc finger, CCCH-type (InterPro:IPR000571); BEST Arabidopsis thaliana protein match is: Zinc finger C-x8-C-x5-C-x3-H type family protein (TAIR:AT4G29190.1); Has 721 Blast hits to 690 proteins in 126 species: Archae - 0; Bacteria - 0; Metazoa - 152; Fungi - 6; Plants - 412; Viruses - 0; Other Eukaryotes - 151 (source: NCBI BLink). & (reliability: 498.0) & (original description: no original description) | 287.6  (Oct) | 5.88e-5 |

**Table S7:** Contingency tables between counts of transcripts in six Hess categories (Hess et al., 2016) and four seasonal photoperiod bins. Each cell contains the counts observed for the null distribution (counts for 2,488 genes identified in the Hess et al., study, and identified as circannually variable in this study) and for the specific Hess category.

Crosstabs for “Day Length – Down” transcripts (N = 537)

Cell Contents

|-------------------------|

| N |

| Expected N |

| Chi-square contribution |

| N / Row Total |

| N / Col Total |

| N / Table Total |

|-------------------------|

Total Observations in Table: 3025

| photoperiod_bin

factor | fall_pp | long_pp | short_pp | spring_pp | Row Total |

-------------|-----------|-----------|-----------|-----------|-----------|

null | 284 | 788 | 1033 | 383 | 2488 |

| 291.158 | 741.876 | 1087.318 | 367.648 | |

| 0.176 | 2.868 | 2.713 | 0.641 | |

| 0.114 | 0.317 | 0.415 | 0.154 | 0.822 |

| 0.802 | 0.874 | 0.781 | 0.857 | |

| 0.094 | 0.260 | 0.341 | 0.127 | |

-------------|-----------|-----------|-----------|-----------|-----------|

day_down | 70 | 114 | 289 | 64 | 537 |

| 62.842 | 160.124 | 234.682 | 79.352 | |

| 0.815 | 13.286 | 12.572 | 2.970 | |

| 0.130 | 0.212 | 0.538 | 0.119 | 0.178 |

| 0.198 | 0.126 | 0.219 | 0.143 | |

| 0.023 | 0.038 | 0.096 | 0.021 | |

-------------|-----------|-----------|-----------|-----------|-----------|

Column Total | 354 | 902 | 1322 | 447 | 3025 |

| 0.117 | 0.298 | 0.437 | 0.148 | |

| 0.991 | 16.154***| 15.285***| 3.611 | 36.041****|

-------------|-----------|-----------|-----------|-----------|-----------|

column *P* <= 0.05 (X2 > X2crit0.05, or 7.815): *

column *P* <= 0.01 (X2 > X2crit0.01, or 11.345): **

column *P* <= 0.005 (X2 > X2crit0.005, or 12.838): ***

column *P* <= 0.001 (X2 > X2crit0.001, or 16.266): ****

Pearson's Chi-squared test

------------------------------------------------------------

Chi^2 = 36.04118 d.f. = 3 p = 7.339753e-08

Fisher's Exact Test for Count Data, day_down model

------------------------------------------------------------

Alternative hypothesis: two.sided p = 5.330766e-08

**Table S7:** continued

Crosstabs for “Day Length – Up” transcripts (N = 332)

Cell Contents

|-------------------------|

| N |

| Expected N |

| Chi-square contribution |

| N / Row Total |

| N / Col Total |

| N / Table Total |

|-------------------------|

Total Observations in Table: 2820

| photoperiod_bin

factor | fall_pp | long_pp | short_pp | spring_pp | Row Total |

-------------|-----------|-----------|-----------|-----------|-----------|

null | 284 | 788 | 1033 | 383 | 2488 |

| 280.562 | 853.155 | 966.967 | 387.316 | |

| 0.042 | 4.976 | 4.509 | 0.048 | |

| 0.114 | 0.317 | 0.415 | 0.154 | 0.882 |

| 0.893 | 0.815 | 0.943 | 0.872 | |

| 0.101 | 0.279 | 0.366 | 0.136 | |

-------------|-----------|-----------|-----------|-----------|-----------|

day_up | 34 | 179 | 63 | 56 | 332 |

| 37.438 | 113.845 | 129.033 | 51.684 | |

| 0.316 | 37.288 | 33.792 | 0.360 | |

| 0.102 | 0.539 | 0.190 | 0.169 | 0.118 |

| 0.107 | 0.185 | 0.057 | 0.128 | |

| 0.012 | 0.063 | 0.022 | 0.020 | |

-------------|-----------|-----------|-----------|-----------|-----------|

Column Total | 318 | 967 | 1096 | 439 | 2820 |

| 0.113 | 0.343 | 0.389 | 0.156 | |

| 0.358 | 42.264****| 38.301****| 0.408 | 81.332****|

-------------|-----------|-----------|-----------|-----------|-----------|

column P <= 0.05 (X2 > X2crit0.05, or 7.815): *

column P <= 0.01 (X2 > X2crit0.01, or 11.345): **

column P <= 0.005 (X2 > X2crit0.005, or 12.838): ***

column P <= 0.001 (X2 > X2crit0.001, or 16.266): ****

Pearson's Chi-squared test

------------------------------------------------------------

Chi^2 = 81.33232 d.f. = 3 p = 1.58938e-17

Fisher's Exact Test for Count Data

------------------------------------------------------------

Alternative hypothesis: two.sided p = 4.743513e-18

**Table S7:** continued

Crosstabs for “Temperature – Down” transcripts (N = 363)

Cell Contents

|-------------------------|

| N |

| Expected N |

| Chi-square contribution |

| N / Row Total |

| N / Col Total |

| N / Table Total |

|-------------------------|

Total Observations in Table: 2851

| photoperiod_bin

$factor | fall_pp | long_pp | short_pp | spring_pp | Row Total |

-------------|-----------|-----------|-----------|-----------|-----------|

null | 284 | 788 | 1033 | 383 | 2488 |

| 260.930 | 759.228 | 1048.084 | 419.757 | |

| 2.040 | 1.090 | 0.217 | 3.219 | |

| 0.114 | 0.317 | 0.415 | 0.154 | 0.873 |

| 0.950 | 0.906 | 0.860 | 0.796 | |

| 0.100 | 0.276 | 0.362 | 0.134 | |

-------------|-----------|-----------|-----------|-----------|-----------|

temp_down | 15 | 82 | 168 | 98 | 363 |

| 38.070 | 110.772 | 152.916 | 61.243 | |

| 13.980 | 7.473 | 1.488 | 22.061 | |

| 0.041 | 0.226 | 0.463 | 0.270 | 0.127 |

| 0.050 | 0.094 | 0.140 | 0.204 | |

| 0.005 | 0.029 | 0.059 | 0.034 | |

-------------|-----------|-----------|-----------|-----------|-----------|

Column Total | 299 | 870 | 1201 | 481 | 2851 |

| 0.105 | 0.305 | 0.421 | 0.169 | |

| 16.020***| 8.563*| 1.705| 25.280****| 51.568****|

-------------|-----------|-----------|-----------|-----------|-----------|

column P <= 0.05 (X2 > X2crit0.05, or 7.815): *

column P <= 0.01 (X2 > X2crit0.01, or 11.345): **

column P <= 0.005 (X2 > X2crit0.005, or 12.838): ***

column P <= 0.001 (X2 > X2crit0.001, or 16.266): ****

Pearson's Chi-squared test

------------------------------------------------------------

Chi^2 = 51.56829 d.f. = 3 p = 3.701774e-11

Fisher's Exact Test for Count Data

------------------------------------------------------------

Alternative hypothesis: two.sided p = 2.218094e-11

**Table S7:** continued

Crosstabs for “Temperature – Up” transcripts (N = 295)

|-------------------------|

| N |

| Expected N |

| Chi-square contribution |

| N / Row Total |

| N / Col Total |

| N / Table Total |

|-------------------------|

Total Observations in Table: 2783

| photoperiod_bin

factor | fall_pp | long_pp | short_pp | spring_pp | Row Total |

-------------|-----------|-----------|-----------|-----------|-----------|

null | 284 | 788 | 1033 | 383 | 2488 |

| 308.430 | 813.539 | 1000.385 | 365.646 | |

| 1.935 | 0.802 | 1.063 | 0.824 | |

| 0.114 | 0.317 | 0.415 | 0.154 | 0.894 |

| 0.823 | 0.866 | 0.923 | 0.936 | |

| 0.102 | 0.283 | 0.371 | 0.138 | |

-------------|-----------|-----------|-----------|-----------|-----------|

temp_up | 61 | 122 | 86 | 26 | 295 |

| 36.570 | 96.461 | 118.615 | 43.354 | |

| 16.320 | 6.762 | 8.968 | 6.947 | |

| 0.207 | 0.414 | 0.292 | 0.088 | 0.106 |

| 0.177 | 0.134 | 0.077 | 0.064 | |

| 0.022 | 0.044 | 0.031 | 0.009 | |

-------------|-----------|-----------|-----------|-----------|-----------|

Column Total | 345 | 910 | 1119 | 409 | 2783 |

| 0.124 | 0.327 | 0.402 | 0.147 | |

| 18.255****| 7.564| 10.031*| 7.771| 43.619****|

-------------|-----------|-----------|-----------|-----------|-----------|

column P <= 0.05 (X2 > X2crit0.05, or 7.815): *

column P <= 0.01 (X2 > X2crit0.01, or 11.345): **

column P <= 0.005 (X2 > X2crit0.005, or 12.838): ***

column P <= 0.001 (X2 > X2crit0.001, or 16.266): ****

Pearson's Chi-squared test

------------------------------------------------------------

Chi^2 = 43.61993 d.f. = 3 p = 1.817481e-09

Fisher's Exact Test for Count Data

------------------------------------------------------------

Alternative hypothesis: two.sided p = 3.039866e-09

**Table S7:** continued

Crosstabs for “Total Available Water – Down” transcripts (N = 271)

Cell Contents

|-------------------------|

| N |

| Expected N |

| Chi-square contribution |

| N / Row Total |

| N / Col Total |

| N / Table Total |

|-------------------------|

Total Observations in Table: 2759

| df4$photoperiod_bin

df4$factor | fall_pp | long_pp | short_pp | spring_pp | Row Total |

-------------|-----------|-----------|-----------|-----------|-----------|

null | 284 | 788 | 1033 | 383 | 2488 |

| 317.425 | 816.107 | 999.168 | 355.300 | |

| 3.520 | 0.968 | 1.146 | 2.160 | |

| 0.114 | 0.317 | 0.415 | 0.154 | 0.902 |

| 0.807 | 0.871 | 0.932 | 0.972 | |

| 0.103 | 0.286 | 0.374 | 0.139 | |

-------------|-----------|-----------|-----------|-----------|-----------|

taw_down | 68 | 117 | 75 | 11 | 271 |

| 34.575 | 88.893 | 108.832 | 38.700 | |

| 32.314 | 8.887 | 10.517 | 19.827 | |

| 0.251 | 0.432 | 0.277 | 0.041 | 0.098 |

| 0.193 | 0.129 | 0.068 | 0.028 | |

| 0.025 | 0.042 | 0.027 | 0.004 | |

-------------|-----------|-----------|-----------|-----------|-----------|

Column Total | 352 | 905 | 1108 | 394 | 2759 |

| 0.128 | 0.328 | 0.402 | 0.143 | |

| 35.834****| 9.855*| 11.660*| 21.987****| 79.338****|

-------------|-----------|-----------|-----------|-----------|-----------|

column P <= 0.05 (X2 > X2crit0.05, or 7.815): *

column P <= 0.01 (X2 > X2crit0.01, or 11.345): **

column P <= 0.005 (X2 > X2crit0.005, or 12.838): ***

column P <= 0.001 (X2 > X2crit0.001, or 16.266): ****

Statistics for All Table Factors, taw_dwn

Pearson's Chi-squared test

------------------------------------------------------------

Chi^2 = 79.33804 d.f. = 3 p = 4.256165e-17

Fisher's Exact Test for Count Data

------------------------------------------------------------

Alternative hypothesis: two.sided p = 2.562889e-17

**Table S7:** continued

Crosstabs for “Total Available Water – Up” transcripts (N = 480)

|-------------------------|

| N |

| Expected N |

| Chi-square contribution |

| N / Row Total |

| N / Col Total |

| N / Table Total |

|-------------------------|

Total Observations in Table: 2968

| df5$photoperiod_bin

df5$factor | fall_pp | long_pp | short_pp | spring_pp | Row Total |

-------------|-----------|-----------|-----------|-----------|-----------|

null | 284 | 788 | 1033 | 383 | 2488 |

| 248.968 | 747.741 | 1091.434 | 399.857 | |

| 4.929 | 2.168 | 3.128 | 0.711 | |

| 0.114 | 0.317 | 0.415 | 0.154 | 0.838 |

| 0.956 | 0.883 | 0.793 | 0.803 | |

| 0.096 | 0.265 | 0.348 | 0.129 | |

-------------|-----------|-----------|-----------|-----------|-----------|

taw_up | 13 | 104 | 269 | 94 | 480 |

| 48.032 | 144.259 | 210.566 | 77.143 | |

| 25.551 | 11.235 | 16.216 | 3.684 | |

| 0.027 | 0.217 | 0.560 | 0.196 | 0.162 |

| 0.044 | 0.117 | 0.207 | 0.197 | |

| 0.004 | 0.035 | 0.091 | 0.032 | |

-------------|-----------|-----------|-----------|-----------|-----------|

Column Total | 297 | 892 | 1302 | 477 | 2968 |

| 0.100 | 0.301 | 0.439 | 0.161 | |

| 30.480****| 13.403***| 19.344****| 4.395 | 67.621****|

-------------|-----------|-----------|-----------|-----------|-----------|

column P <= 0.05 (X2 > X2crit0.05, or 7.815): *

column P <= 0.01 (X2 > X2crit0.01, or 11.345): **

column P <= 0.005 (X2 > X2crit0.005, or 12.838): ***

column P <= 0.001 (X2 > X2crit0.001, or 16.266): ****

Pearson's Chi-squared test

------------------------------------------------------------

Chi^2 = 67.6216 d.f. = 3 p = 1.378553e-14

Fisher's Exact Test for Count Data

------------------------------------------------------------

Alternative hypothesis: two.sided p = 1.538449e-16
